# Disruption of neural periodicity predicts clinical response after deep brain stimulation for obsessive-compulsive disorder

**DOI:** 10.1038/s41591-024-03125-0

**Published:** 2024-07-12

**Authors:** Nicole R. Provenza, Sandesh Reddy, Anthony K. Allam, Sameer V. Rajesh, Nabeel Diab, Gabriel Reyes, Rose M. Caston, Kalman A. Katlowitz, Ajay D. Gandhi, Raphael A. Bechtold, Huy Q. Dang, Ricardo A. Najera, Nisha Giridharan, Katherine E. Kabotyanski, Faiza Momin, Mohammed Hasen, Garrett P Banks, Brian J. Mickey, Brent M. Kious, Ben Shofty, Benjamin Y. Hayden, Jeffrey A. Herron, Eric A. Storch, Ankit B. Patel, Wayne K. Goodman, Sameer A. Sheth

**Affiliations:** 1 Department of Neurosurgery, Baylor College of Medicine, Houston, TX, USA.; 2 Department of Electrical & Computer Engineering, Rice University, Houston, TX, USA.; 3 Department of Neurosurgery, University of Utah, Salt Lake City, UT, USA.; 4 Department of Bioengineering, University of Washington, Seattle, WA, USA.; 5 Department of Psychiatry, University of Utah, Salt Lake City, UT, USA.; 6 Department of Biomedical Engineering, University of Utah, Salt Lake City, UT, USA.; 7 Department of Neuroscience, Baylor College of Medicine, Houston, TX, USA.; 8 Department of Neurological Surgery, University of Washington, Seattle, WA, USA.; 9 Menninger Department of Psychiatry and Behavioral Sciences, Baylor College of Medicine, Houston, TX, USA.; 10 These authors contributed equally: Nicole R. Provenza, Sandesh Reddy, Anthony K. Allam.

## Abstract

Recent advances in surgical neuromodulation have enabled chronic and continuous intracranial monitoring during everyday life. We used this opportunity to identify neural predictors of clinical state in 12 individuals with treatment-resistant obsessive-compulsive disorder (OCD) receiving deep brain stimulation (DBS) therapy (NCT05915741). We developed our neurobehavioral models based on continuous neural recordings in the region of the ventral striatum in an initial cohort of five patients and tested and validated them in a held-out cohort of seven additional patients. Before DBS activation, in the most symptomatic state, theta/alpha (9 Hz) power evidenced a prominent circadian pattern and a high degree of predictability. In patients with persistent symptoms (non-responders), predictability of the neural data remained consistently high. On the other hand, in patients who improved symptomatically (responders), predictability of the neural data was significantly diminished. This neural feature accurately classified clinical status even in patients with limited duration recordings, indicating generalizability that could facilitate therapeutic decision-making.

Conventional approaches to diagnosing and monitoring outcomes for psychiatric disorders have relied on categorizing symptom phenomenology, a scheme that has provided standardization to the field of psychiatry and facilitated communication among its practitioners over the past half century. High on the list of modern era criticisms, however, is its inherently phenomenological nature, focused on statistical patterns of symptom clusters rather than on underlying cause and mechanism. The past decade has evidenced an accelerating interest in developing a mechanistically oriented framework within which to study these disorders and assess their treatments. Most widely adopted of these is the National Institute of Mental Health’s Research Domain Criteria (RDoC)^1^, a trans-diagnostic framework defined by fundamental neurobehavioral constructs rather than symptom clusters.

In the present study, we focused on a pair of related and well-studied constructs within this framework—response selection and inhibitory control—which lie within the broader category of cognitive control. In the context of decision-making, these terms refer to the ability to identify environmental or internal factors relevant to a pending decision, ignore irrelevant factors and arrive at an optimal decision^2,3^. They also refer to the ability to suppress pre-potent, automatic responses in favor of reasoned ones that may be more adaptive in the longer term^4^. These factors, therefore, reflect a latent axis, or continuum, of phenotypes. As with most axes defined in such behavioral terms, an adaptive regime typically resides in the middle ground, a balance between the extremes, with the capacity to adjust the dial according to situational demands. Extrema along this axis, however, constitute well-characterized mental illnesses. On one end lie unbridled ‘approach’ behaviors and their attendant disorders of impulsiveness. On the other lie ‘avoidant’ phenotypes, marked by disproportional fear and anxiety^5–7^.

Obsessive-compulsive disorder (OCD) is a classic example of the latter extreme. We^8^ and others^9^ have highlighted dysfunctional response selection as a central feature in the pathophysiology of OCD. OCD is common and debilitating, with a prevalence of 2–3% in the general population^10^. In the most severe cases, patients spend an extraordinary amount time performing repetitive, seemingly senseless compulsions and perseverating on distressful, intrusive thoughts. Despite having insight that the thoughts are irrational and the compulsions unproductive, these individuals feel an overwhelming urge to continue these behaviors. Unlike disorders of addiction in which behavior is reward seeking, OCD-related compulsions are difficult-to-control avoidant responses to internal thoughts or external triggers that develop to evade distress or harm^5,6^. The avoidant diathesis is more obvious in certain subtypes of OCD, such as the contamination subtype (that is, avoiding germs by washing hands), but it also characterizes others. For example, individuals with taboo thoughts avoid saying or thinking certain things lest doing so causes harm to someone; those with the ‘just right’ subtype are compelled to repeat actions until performed ‘correctly’ to avoid a negative consequence of performing them ‘incorrectly’.

Although pathological avoidance is not the only form of cognitive dysfunction in OCD, a substantial literature supports the idea that OCD symptomatology projects appreciably onto the axis of pathological avoidance and motivates our focus on this behavioral dimension. Greater degrees of avoidance are associated with greater OCD severity^7^ and greater treatment resistance^11^. Exposure and response prevention (ERP) therapy is an effective, evidence-based treatment built on the premise that obsessive-compulsive symptoms are a manifestation of an avoidant phenotype and that overcoming that avoidance (facilitating approach) can improve symptoms^12^. During ERP, a therapist guides the patient through interactions with OCD triggers (that is, exposures) that elicit distress while encouraging the patient to not engage in their typical attendant compulsions. Over time, engaging in the systematic approach toward and tolerance of distress-evoking triggers facilitates formation of new non-threatening associations, leading to symptom improvement^13^.

For the 10–20% of individuals with OCD who are treatment resistant, deep brain stimulation (DBS) of the ventral striatum (VS) and adjacent capsular white matter (ventral capsule (VC)) is an effective therapy, with response rates in the 66% range^14^ and regulatory approval in the form of a humanitarian device exemption from the US Food & Drug Administration. Whereas improvement in OCD symptoms per se can take months to manifest, initiation of stimulation can have immediate and profound effects on mood and energy. These acute effects of VC/VS DBS activation have been labeled ‘mirth’ or ‘positive affect’ responses, variably consisting of smiling (often a contralateral hemi-smile), laughter or feelings of increased energy^15–18^. The early appearance of these behaviors is a possible predictor of eventual clinical response^18,19^, although notable counter-examples exist^16^. Based on our experience with this cohort and others^19–22^, we adopted the more general terminology of ‘approach’ behavior to characterize this response, as one of the most common observable changes is an increase in talkativeness, a desire to engage in activities and extroversion. Indeed, a common side effect of overstimulation is the induction of overly approachful, disinhibited (often termed hypomanic^15–18^) behavior, characterized by impulsivity, overabundance of energy, increased libido and decreased need for sleep.

We, thus, focused on the approach-avoidance axis as one of the critical neurobehavioral axes underlying the pathophysiology of OCD. Although certainly not the only pathological axis in OCD, it does capture important behavioral manifestations of the disorder. To better understand its neurophysiological basis, we sought to identify neural signatures of transitions along this behavioral axis produced by VC/VS DBS. Doing so would further anchor key diagnostic features within this cognitive framework, help advance its mechanistic understanding and, ideally, influence monitoring and treatment strategies.

To accomplish this goal, we took advantage of a novel opportunity for continuous long-term intracranial recordings in patients with OCD. Recently available DBS systems have the ability to not only deliver stimulation but also record local field potentials (LFPs)^20^. Low-frequency oscillations in the theta (4–8 Hz) to alpha (8–12 Hz) range play a prominent role in the large body of neuroscientific literature on cognitive control, from animal studies^23,24^ to human non-invasive^25,26^ and intracranial^27,28^ neurophysiology. As opposed to using traditional episodic, task-based cognitive neurophysiology approaches, we adopted the more ethologically relevant approach made available with on-device recordings to determine whether variations in continuously acquired neural data could provide an objective readout of clinical state transitions (that is, from the avoidant state that characterizes severe OCD symptoms to the more approachful, tolerant state that characterizes clinical response).

We hypothesized that changes in the periodicity of neural signals may provide insight into pathological network activity and, therefore, clinical status. Abnormalities in daily (circadian) periodicity are a cardinal feature of mental health disorders in general^29^ and of OCD in particular^30,31^. Several studies identified a relationship between OCD symptom severity and circadian pattern alteration, including tendencies for delayed sleep onset and/or insomnia^30,31^. Given the frequent sampling required to identify circadian neural variations^32–34^, as well as the extended time period required to study the clinical trajectory of response to DBS (months)^35^, we leveraged the long-term recording capability of these DBS systems to track monthsʼ worth of low-frequency power changes in 12 individuals with severe, treatment-resistant OCD. Our results demonstrate that 9-Hz (theta/alpha border) VS neural activity is highly periodic in the symptomatic state. Decreased periodicity and predictability of this signal after DBS initiation characterized clinical response, whereas persistence of these features marked non-response.

## Results

**Fig. 1 |:**
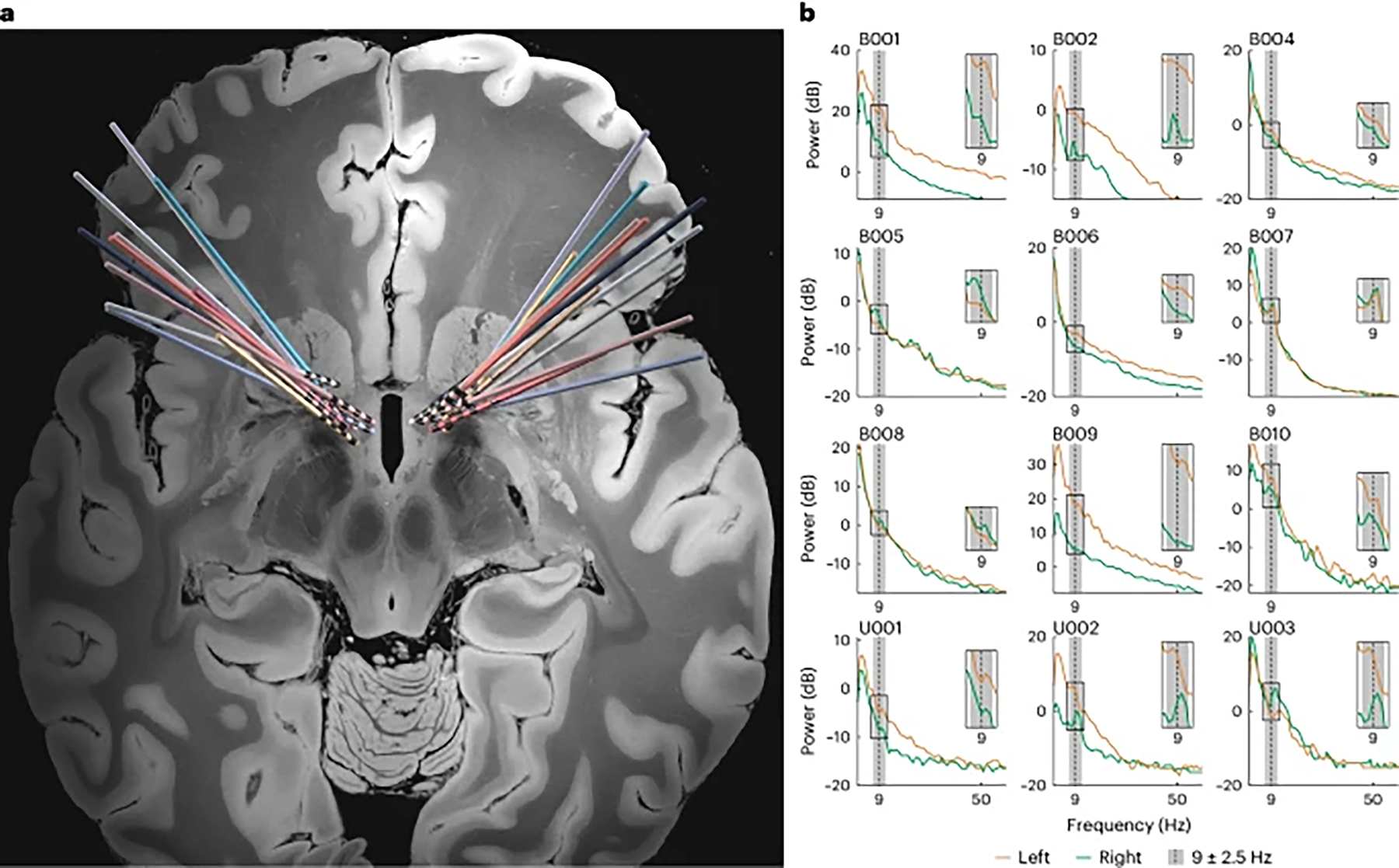
VS recordings in patients with OCD show narrow band ~9 Hz power feature. **a**, Reconstruction of DBS lead placement in VC/VS (*n* = 12) in standard atlas space. **b**, Power spectral density plots for each patient (green: left hemisphere; dark yellow: right hemisphere). In some cases, we were only able to acquire recordings from one hemisphere due to stimulation configuration. Insets zoom in on 9 Hz, marked by the vertical dotted line. Gray shaded region shows the frequency band that we configured for chronic recordings (9 ± 2.5 Hz).

Twelve patients with severe OCD who met surgical criteria for DBS participated in this study (NCT05915741). We bilaterally implanted DBS leads in the region of the VC/VS as previously described^19^ (Fig. 1a). We also implanted a Medtronic Percept PC pulse generator, which not only delivers stimulation but can also record neural activity. Of the 12 patients, eight had clinical follow-up for more than 6 months (mean follow-up duration: 22.75 ± 8.4 months). Of those eight, five (62.5%) were responders to DBS (Yale-Brown Obsessive-Compulsive Scale (Y-BOCS) reduction ≥ 35%) (Table 1).

The 12 patients span two temporally contiguous cohorts. Cohort 1 consists of the first five patients (B001–B006), from whom we have the longest recordings, starting before DBS initiation and spanning months afterwards until they achieved stable chronic clinical status. Data from these patients were used to identify promising models linking neural activity to clinical state. Cohort 2 consists of the next seven patients (B007–U003), whose data were more limited for several reasons (Methods). We performed predictive testing on this expanded cohort to identify generalizable neurobehavioral relationships across patients.

### VS low-frequency power shows circadian periodicity before DBS

In the first few patients in Cohort 1, we noticed a narrow-band peak in VS spectral power at the theta/alpha border (at or near 9 Hz) (Fig. 1b). Given the prominence of this neural feature and its role in cognitive neurophysiology, we chose 9 Hz as the center frequency to track upon implantation of the DBS system. We, thus, obtained chronic, passive recordings in this band at continuous 10-min intervals in all patients. The duration of recordings amounted to several months’ worth in each of the five Cohort 1 patients (316 ± 128 d total duration per patient; 25 ± 20 d before VS DBS activation and 292 ± 136 d after) and several weeks’ worth in each of the seven Cohort 2 patients (67 ± 46 d total duration per patient).

**Fig. 2 |:**
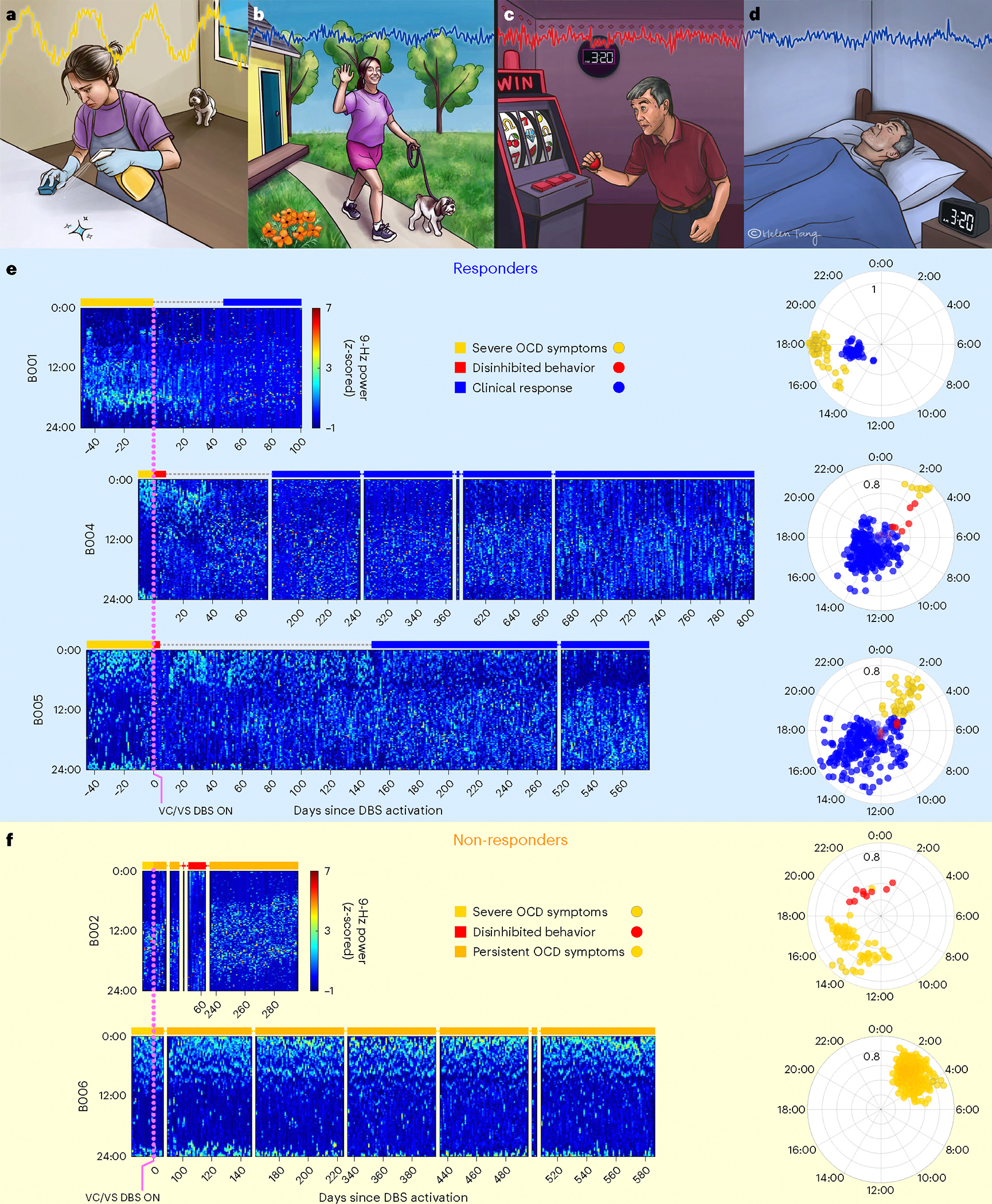
Temporal dynamics of VS neural activity reflect behavioral phenotype and clinical status. **a**–**d**, Illustration depicting prototypical clinical/behavioral states associated with OCD and treatment with DBS. **a**, Avoidant rituals of the severely symptomatic state. **b**, Adaptive behaviors of clinical response. **c**, Excessively approachful behaviors of overstimulation, including disinhibition and decreased need for sleep. **d**, Balanced activities and sleep/wake patterns characteristic of clinical response. **e**, Left, heatmaps of left hemisphere VS 9-Hz power (color bar) versus time of day (*y* axis) and days since VC/VS DBS activation (*x* axis) in the three clinical responders from Cohort 1. Colored bars over heatmaps indicate behavioral/clinical state. Right, polar plots with cosinor fit amplitude versus acrophase over time. The circadian pattern amplitude clearly distinguishes among the symptomatic state (light yellow; strong circadian pattern), the overly disinhibited state (red; abolished circadian pattern) and clinically stable response (blue; reduced circadian pattern). **f**, Same as **e** but for non-responders. There is less separation between the pre-DBS (light yellow) and long-term non-response (dark yellow) states.

By collecting these continuous recordings, we sought to identify neural biomarkers of clinical states relevant to this disorder. One important state is that of severe OCD symptom burden, which describes the initial state for all patients before DBS activation. In the context of our approach-avoidance framework, this is a state marked by highly avoidant behaviors performed in response to exaggerated or irrational representations of threat (Fig. 2a). At the other extreme of the behavioral spectrum is a less common clinical state that can occur as a side effect of VC/VS DBS. This state consists of an overly approachful phenotype marked by notably disinhibited behavior (for example, risk taking, increased libido, impulsivity and overabundance of energy) (Fig. 2c)^15,16,18^. Between these two extremes lies the state of clinical response, which represents an adaptive balance between these behavioral poles (Fig. 2b,d). More than 48,000 h of recordings in these patients demonstrated a remarkable correspondence between neural activity and clinical status.

**Extended Data Fig. 1 |:**
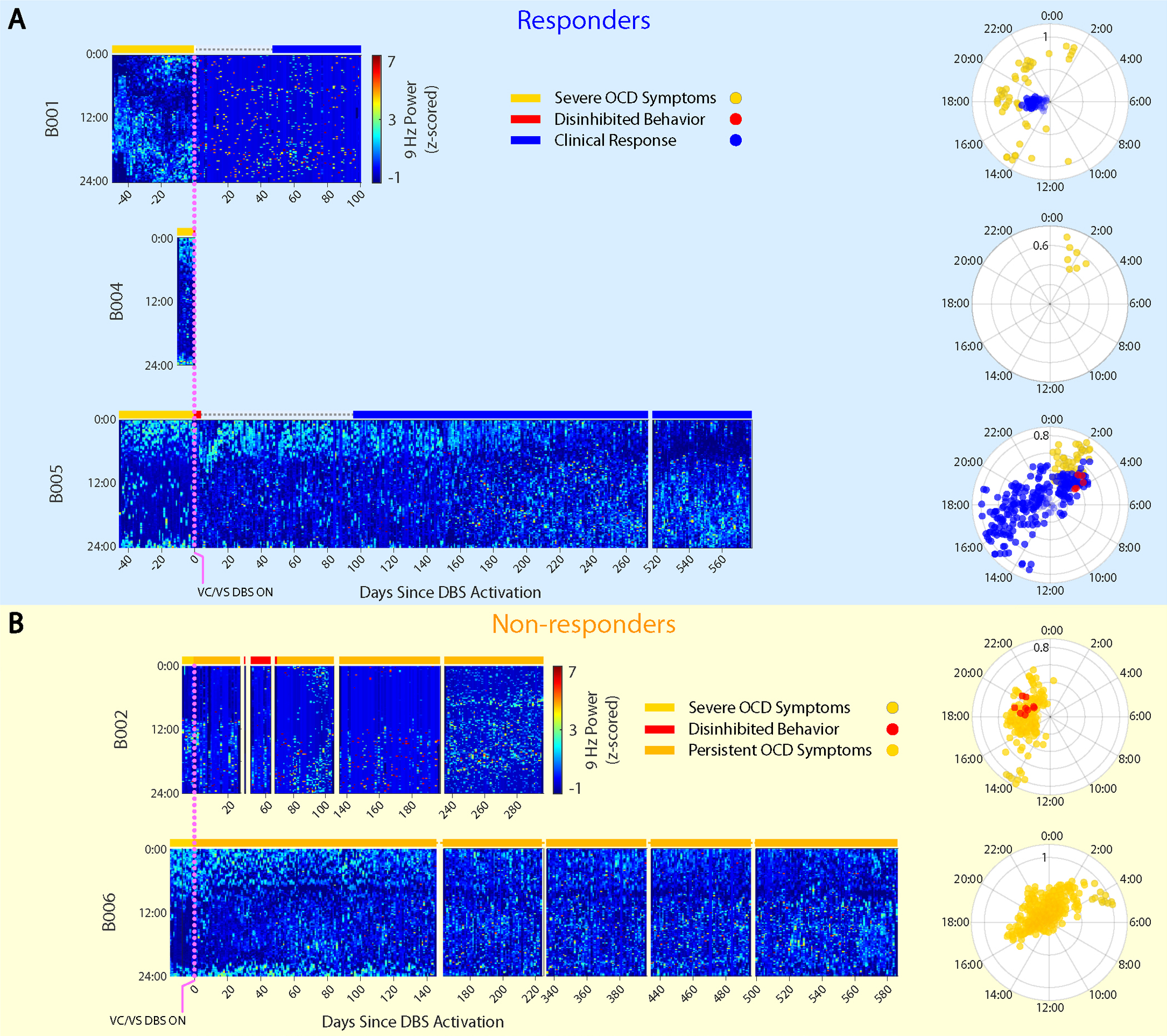
Temporal dynamics of ventral striatum neural activity reflects behavioral phenotype and clinical status. (**a**) (left), Heatmaps of right hemisphere VS 9 Hz power (colorbar) vs. time of day (y-axis) and days since VC/VS DBS activation (x-axis) in the three clinical responders from Cohort 1. Colored bars over heatmaps indicate behavioral/clinical state. (right), Polar plots with cosinor fit amplitude vs. acrophase over time. The amplitude of the circadian pattern clearly distinguishes between the severely symptomatic state (light yellow with grey outline; strong circadian pattern), overly disinhibited state (red; abolished circadian pattern), and clinically stable response (blue; reduced circadian pattern). (**b**), Same as in (**A**) but for non-responders. There is less separation between the pre-DBS symptomatic state (light yellow with grey outline) and the state of long-term non-response (dark yellow).

Because LFP recordings are enabled immediately upon implant, but stimulation typically starts weeks later, we were able to obtain neural recordings for the baseline symptomatic clinical state. As evident from the heatmaps showing 9-Hz spectral power in the five Cohort 1 patients in Fig. 2e,f (left hemisphere) and Extended Data Fig. 1a,b (right hemisphere), this symptomatic state before DBS activation demonstrated a characteristic cyclic pattern of 9-Hz power with an approximately daily period.

We applied four metrics (three model-based and one model-free) to assess this periodicity and its change after DBS activation. We did so in the Cohort 1 patients first, because of their more extensive data. Given the circadian-appearing neural activity pattern, we began by applying a cosinor regression model, a common model used for measuring circadian periodicity in biological systems. Modeling demonstrated a significant circadian (24-h period) fit in both the left (*P* < 10^–63^; Fig. 2e,f and Supplementary Table 1) and the right (*P* < 10^–44^; Extended Data Fig. 1a,b and Supplementary Table 1) hemispheres. This circadian periodicity of 9-Hz neural activity was consistent across patients before DBS activation (Fig. 2e,f and Extended Data Fig. 1a,b).

DBS initiation elicited notable behavioral and neurophysiological changes. In four of the five Cohort 1 patients (B001, B002, B004 and B005), we observed acute pro-approach effects upon DBS initiation. The pro-approach response was potent enough in two patients (B004 and B005) that they exhibited clinically meaningful and maladaptive disinhibited behavior in the days immediately after DBS activation (red bars in Fig. 2e,f). In these two patients, the neural recordings demonstrated changes mirroring these behavioral observations. There was a reduction in amplitude of the circadian neural pattern that is evident in the polar plots in Fig. 2e,f as proximity of the red points to the origin. DBS programming adjustments (that is, decreased amplitude) at the next clinic visit alleviated those behaviors. Because of the short duration of these disinhibited periods and dearth of reliable behavioral data, we did not attempt to model these acute effects.

### Loss of VS neural predictability indicates clinical response

We focused our efforts on identifying neural predictors of long-term clinical state (that is, responder versus non-responder status). We classified ‘clinical response’ as time periods when a patient demonstrated clinically meaningful improvement in OCD symptoms and periods of ‘persistent OCD symptoms’ based on analogous opposite criteria (Methods). The heatmaps of 9-Hz power demonstrated a marked reduction in circadian periodicity during periods of clinical response relative to the pre-DBS symptomatic state (Fig. 2e,f and Extended Data Fig. 1a,b). Consistently, the amplitude of the cosinor fit was reduced during these periods (Fig. 2e,f, polar plots).

**Fig. 3 |:**
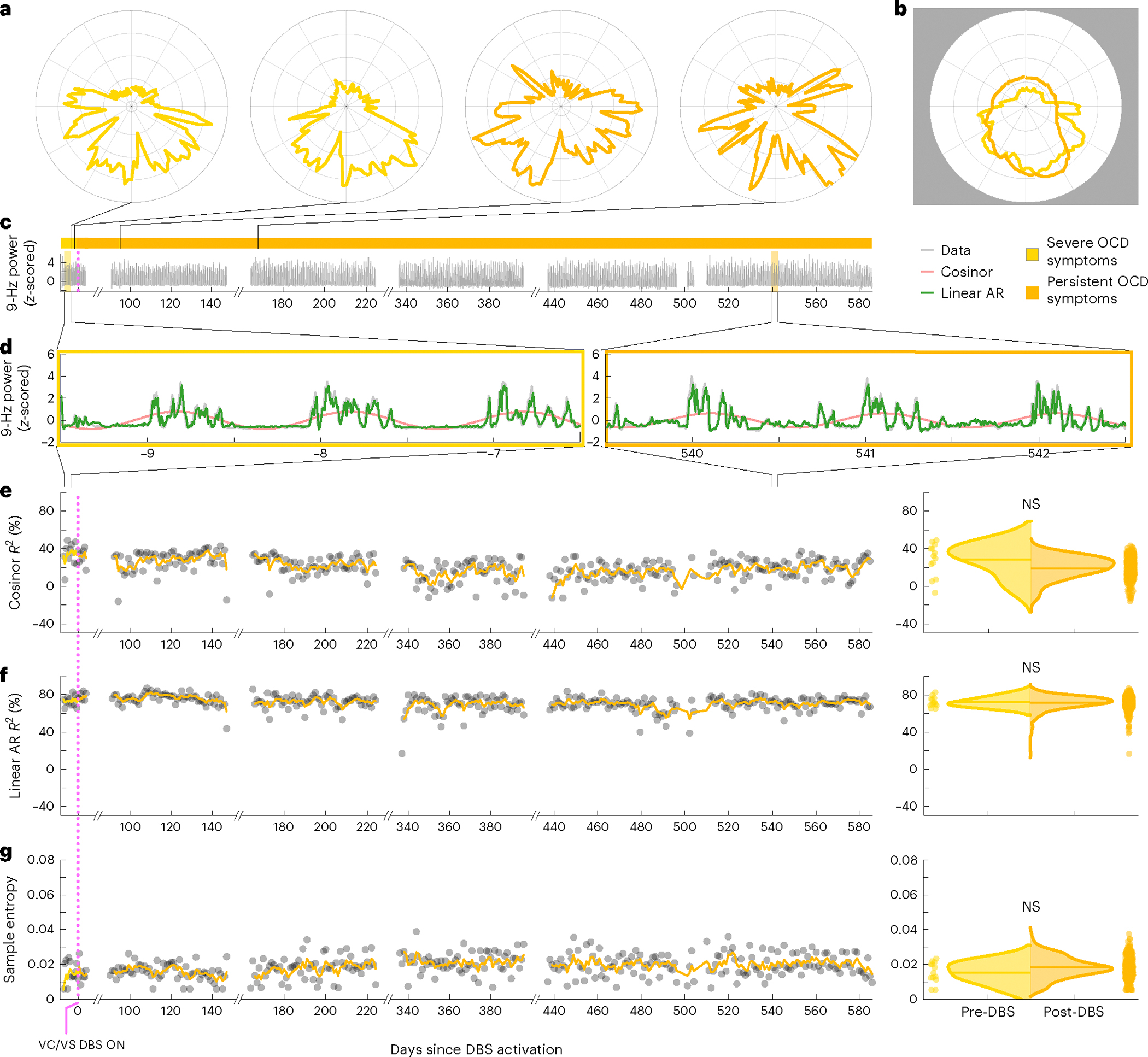
VS neural activity is highly circadian and predictable in the severe OCD symptom state. All figure panels were constructed using data from one patient as an example non-responder, B006. **a**, Circularized plots of daily neural activity patterns showing *z*-scored 9-Hz power (radial axis) versus time of day (angular axis). The first two plots show representative days from the pre-DBS period, and the second two plots show representative days from the post-DBS period. The data within each day were rotated to align the peak daily amplitude to 3π/2 to allow averaging across days. **b**, Circularized plot showing the average daily neural activity pattern over the entire pre-DBS period in light yellow and the post-DBS period in dark yellow. **c**, *z*-scored 9-Hz power over days throughout the entire monitoring period. **d**, Callouts of normalized 9-Hz power and model fits showing 3-d periods before DBS (left) and after DBS (right). We fit a cosinor model (pink) and linear autoregressive model (green) to the raw data (gray). The linear autoregressive model was better able to capture intra-day fluctuations in 9-Hz power than was the cosinor model. The nonlinear model fit is not depicted as it was visually indistinguishable from the linear model fit. Daily cosinor $R^{2}$ (**e**), linear autoregressive $R^{2}$ (**f**) and sample entropy values (gray) (**g**) are plotted over time and overlaid by a 5-d exponential moving average (EMA) line (left). The color of the EMA line over time reflects clinical status as described in **b**. The half-violin plots (right) indicate the distribution of daily values within each clinical state. The daily values are not significantly different before versus after DBS activation in any of the metrics, via a two-tailed Welch’s *t*-test without multiple comparisons. Respective *P* values are 0.066, 0.510 and 0.772. AR, autoregressive; NS, not significant.

**Fig. 4 |:**
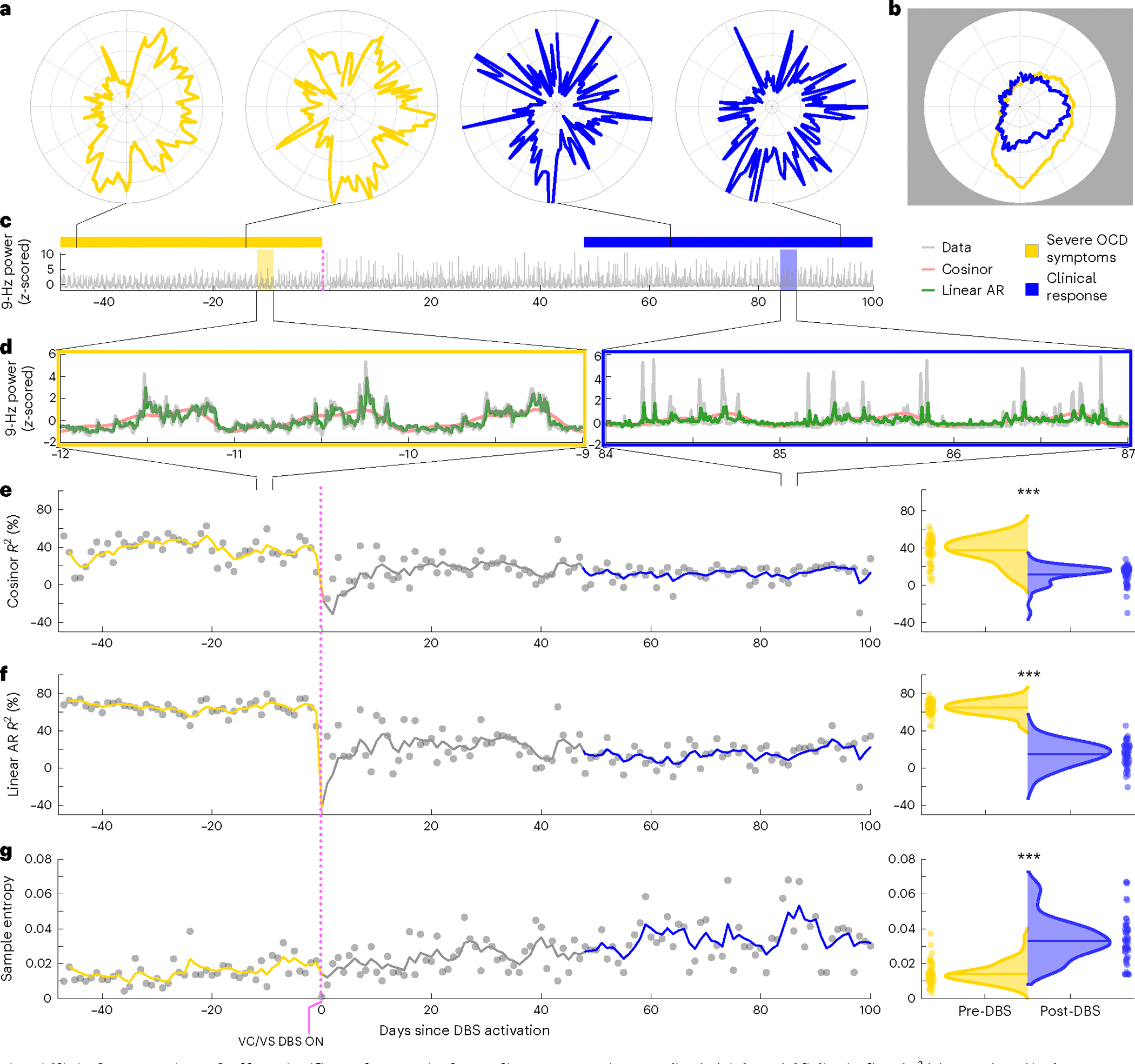
Clinical response is marked by a significant decrease in the predictability of VS neural activity. All figure panels were constructed using data from one patient as an example responder, B001. **a**, Same schema as in Fig. 3a, showing two representative days from the pre-DBS period (light yellow) and two from the post-DBS responder period (blue). **b**, Circularized plot showing the average daily neural activity pattern over the entire pre-DBS symptomatic period (light yellow) and the post-DBS responder period (blue). **c**–**g**, Same schema as in Fig. 3c–g. As opposed to the close fits in the non-responder data in Fig. 3, the statistical metrics do not fit the post-DBS period well in this clinical responder. Cosinor amplitude (pink model fit line in **d**) and $R^{2}$ (**e**) are reduced in the post-DBS versus pre-DBS data. The linear autoregressive model (green line in **d**) does not match the raw data (gray line in **d**) as well as it did in Fig. 3, and the model $R^{2}$ (**f**) is also reduced in the post-DBS versus pre-DBS data. Consistently, sample entropy increases over time. All three metric outputs (half-violin plots in **e**–**g**) are significantly different between the pre-DBS and post-DBS periods (asterisks), via a two-tailed Welch’s *t*-test without multiple comparisons. Respective *P* values are 1.02 × 10^−16^, 2.46 × 10^−40^ and 4.10 × 10^−14^. AR, autoregressive.

To study this effect, we created circularized measures of daily neural activity patterns (Figs. 3a and 4a). These circularized plots demonstrate several notable features. The circadian periodicity is evident as a lobed appearance or eccentricity in the daily circular plots of the pre-DBS (Fig. 3a, left panels, and Fig. 4a, left panels; light yellow) and non-responder (Fig. 3a, right panels; dark yellow) states and even more so in the state-averaged circularized plots (light and dark yellow in Figs. 3b and 4b). In contrast, the circularized plots of responder status (Fig. 4a, right panels; blue) show less eccentricity and circadian periodicity. This effect is also most evident in the state-averaged plot (Fig. 4b). To illustrate these effects, we created time-lapse animations of daily circularized plots, which, in responders (Supplementary Video 1), demonstrate the transition from patterns primarily characterized by eccentricity (strong circadian component) to ones with a prominent stellate shape (intra-day dispersion of neural power). In contrast, in non-responders, the pre-DBS eccentric pattern does not appreciably change (Supplementary Video 2).

We quantified the change in strength of circadian periodicity before versus after DBS by comparing the cosinor model goodness of fit ($R^{2}$) between the two states. Based on the observations above, we expected a significant decrease in $R^{2}$ (indicating decrement in periodicity) in clinical responders compared to no significant change in $R^{2}$ (indicating persistent periodicity) in non-responders. After accounting for autocorrelation in the data arising from frequent neural sampling (every 10 min) but lack of frequent variation in clinical status (responder versus non-responder) labels, the cosinor model performed relatively poorly under these conditions, with results consistent with expectations in only two of five hemispheres in clinical responders and in three of four hemispheres in non-responders (Extended Data Table 1). Without sample size correction, the cosinor model performed better, with consistent results in five of five responders and in three of four non-responders (Extended Data Table 1).

Moderate performance and the overly simple nature of the cosinor model motivated us to test other models. Besides decreased circadian periodicity, another feature evident in the responder circularized plots is increased daily variability or dispersion of neural activity, visible as spikiness. The cosinor model assumes a sinusoidal shape and is not well suited to capture these additional elements of periodicity. We, thus, used a family of autoregressive models, which do not assume a particular shape of periodic data but simply test whether previous datapoints are predictive of future datapoints. The linear and nonlinear autoregressive models fit the pre-DBS and non-responder neural data much more closely than did the cosinor model (Fig. 3d). Whereas the latter model simply captured the general daily/circadian trend, the former models matched within-day variations in neural activity with greater fidelity.

**Extended Data Fig. 2 |:**
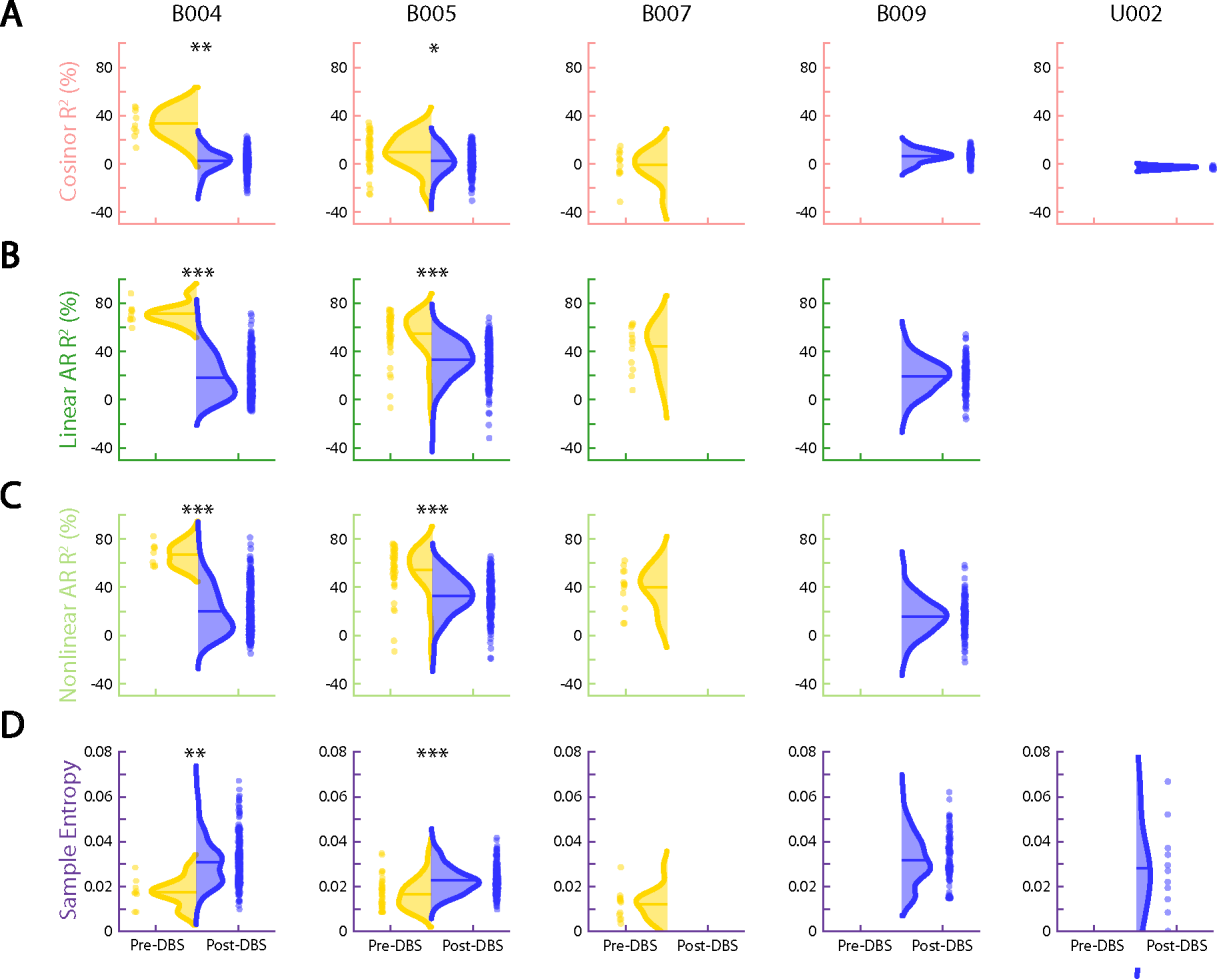
Ventral striatum output measures distinguish pre-DBS severe symptom state from post-DBS clinical response. (**a**) Half-violin plots indicate the distribution of daily cosinor R2 values for the pre-DBS severe OCD symptom state (yellow) and the post-DBS clinical response state (blue). The horizontal line denotes the median of each distribution. Distributions with a statistically significant difference (*p < 0.01, **p < 10−4, ***p < 10−6) between the pre-DBS and clinical response period using two-tailed Welch’s t-tests without multiple comparisons are denoted with an asterisk above the corresponding plot. Panels B, C, and D are formatted identically to Panel A, but show (**b**) daily linear AR R2, (**c**) daily non-linear AR R2, and (**d**) daily sample entropy. U002 does not appear in linear and nonlinear autoregressive R2 estimations due to missing data preventing calculation of the daily output measures. Exact p-values correspond to the top-left quadrant of Extended Data Tables 1–4. **Abbreviations:** R2, coefficient of determination; AR, autoregression.

**Extended Data Fig. 3 |:**
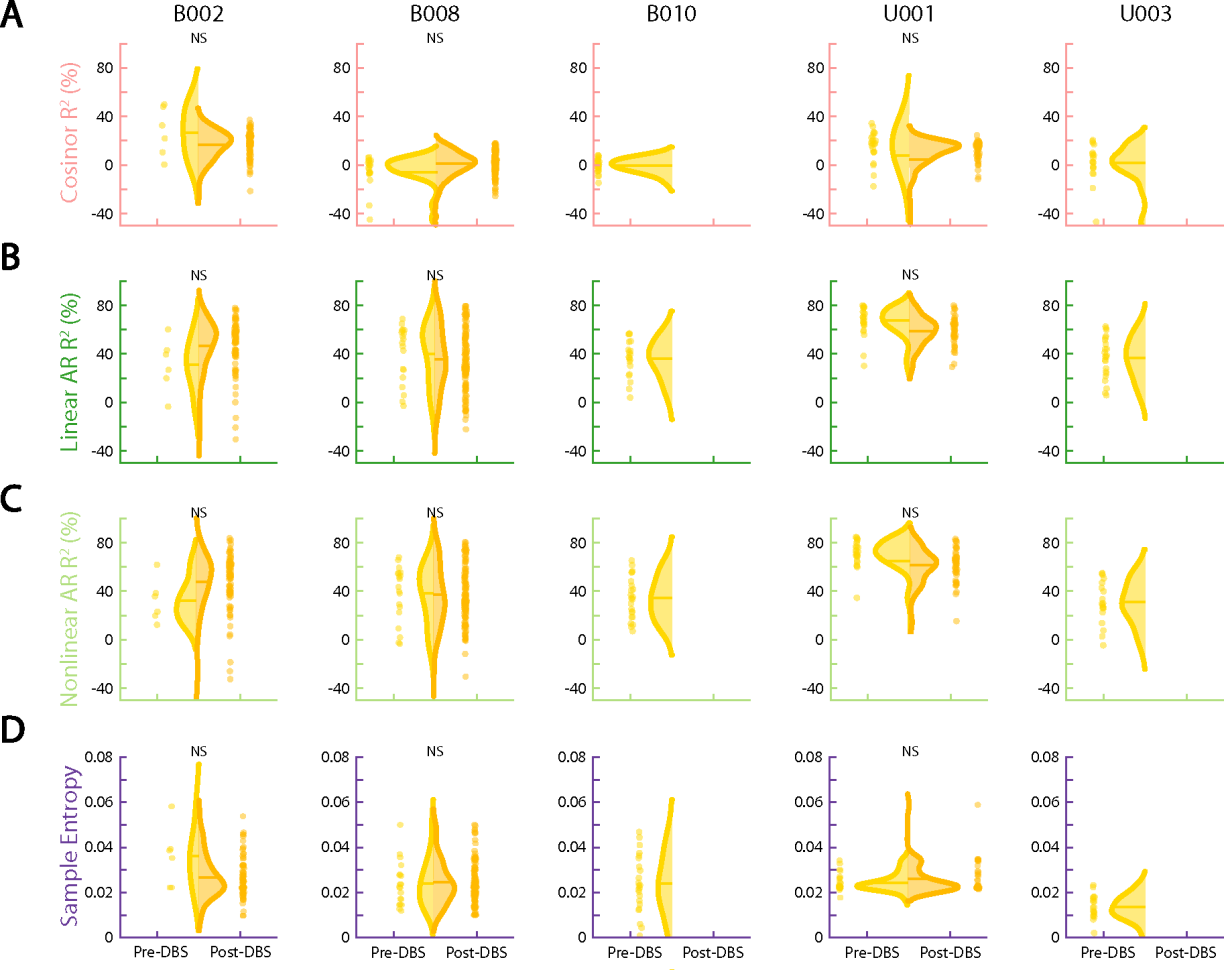
Ventral striatum output measures are indistinguishable before and after DBS in non-responders. (**a**) Half-violin plots indicate the distribution of daily cosinor R2 values for the pre-DBS severe OCD symptom state (yellow) and the post-DBS persistent symptom state state (dark yellow). The horizontal line denotes the median of each distribution. Distributions with a statistically significant difference (*p < 0.01, **p < 10−4, ***p < 10−6) between the pre-DBS and clinical response period are denoted with an asterisk above the corresponding plot. Panels B, C, and D are formatted identically to Panel A, but show (**b**) daily linear AR R2, (**c**) daily non-linear AR R2, and (**d**) daily sample entropy. Exact p-values correspond to the top-left quadrant of Extended Data Tables 1–4 for B002 and B008 and the top-left quadrant of Extended Data Table 5 for U001. **Abbreviations:** R2, coefficient of determination; AR, autoregression.

The close fits of the autoregressive models (Figs. 3 and 4 and Extended Data Figs. 2 and 3) indicate a high degree of predictability of 9-Hz VS neural activity in the pre-DBS symptomatic state. Both linear and nonlinear autoregressive models discriminated clinical response from non-response more consistently than did the cosinor model. Results for these models matched the expected pattern in five of five hemispheres in responders and in three of four hemispheres in non-responders, not only without correction for non-independence but even with the conservative sample size correction (Extended Data Tables 2 and 3). In other words, clinical response was associated with decreased predictability of neural activity, whereas non-response was associated with persistent neural predictability. The sole inconsistency was the right hemisphere of patient B006, in whom the neural pattern looked like that of a responder despite the fact that, clinically, the patient was a non-responder. Examples of hemispheric asymmetries between brain activity and clinical outcomes are common in OCD. Functional imaging studies identified lateralized changes in blood flow^36^, metabolic activity^37^ and functional connectivity^38^ in response to pharmacological, cognitive-behavioral and even DBS therapy. Our data also demonstrate more reliable predictability of clinical status from left VS neural data than from the right, as further demonstrated below for individual patient analyses in Cohort 1 and in the across-patient cross-validated predictive analyses in the full cohort.

In addition to these model-based metrics, we also used sample entropy, a model-free metric, to capture this change in periodicity and variability. Sample entropy quantifies the regularity and (un)predictability of time-series data, making it a good candidate for data with these features. Because it is a computed value rather than a model, we compared its value rather than goodness of fit pre-DBS versus post-DBS. A finding consistent with our expectation would be a significant increase in sample entropy in responders compared to no change in non-responders. Sample entropy performed better than the cosinor model but not as well as the autoregressive models. It behaved as expected in five of five responder hemispheres and in three of four non-responder hemispheres without sample size correction but in only four of five responder and in three of four non-responder hemispheres with the conservative sample size correction (Extended Data Table 4).

### VS predictability indicates response status in full cohort

To determine whether these metrics generalize to a larger sample, we applied them to the seven additional Cohort 2 patients (Extended Data Figs. 2 and 3) who had less data for a variety of reasons (Methods). Extended Data Figs. 2 and 3 show distributions of the four output measures (cosinor and autoregressive $R^{2}$ values and sample entropy) for the 10 remaining patients not shown in Figs. 3 and 4. Three Cohort 2 patients—one responder (B007) and two non-responders (B008 and U001)—had at least one hemisphere’s worth of both pre-DBS and post-DBS data (Extended Data Table 5), but the remaining four Cohort 2 patients only had intervals of pre-DBS or post-DBS data.

We used the combined sample of all 12 patients’ data across both cohorts to test the four metrics for generalizability using cross-validated predictive modeling. To do so, we dichotomized the data into symptom burdened and unburdened states. The symptom burdened state included periods of time before DBS activation (when all patients are symptomatic; light yellow lines in Figs. 2–4) and after DBS activation in clinical non-responders (persistent symptoms; dark yellow lines in Figs. 2–4). The symptom unburdened state simply included post-DBS periods of clinical response (blue lines in Figs. 2–4).

We began by comparing the distributions of each of the four output measures between the symptom burdened and unburdened states (Extended Data Table 6). As we did for Cohort 1 above, we did so both with and without sample size correction to account for non-independence arising from autocorrelation of the clinical state labels. Without correction, the autoregressive models and sample entropy could reliably distinguish clinical state in both left (*P* < 10^−47^) and right (*P* < 10^−17^) hemispheres, but the cosinor model could do so only in the left hemisphere (*P* < 10^−2^) (Extended Data Table 6). With conservative correction, the autoregressive models and sample entropy were still successful in both left (*P* < 10^−15^) and right (*P* < 10^−5^) hemispheres, but the cosinor model was no longer successful in either (Extended Data Table 6).

**Fig. 5 |:**
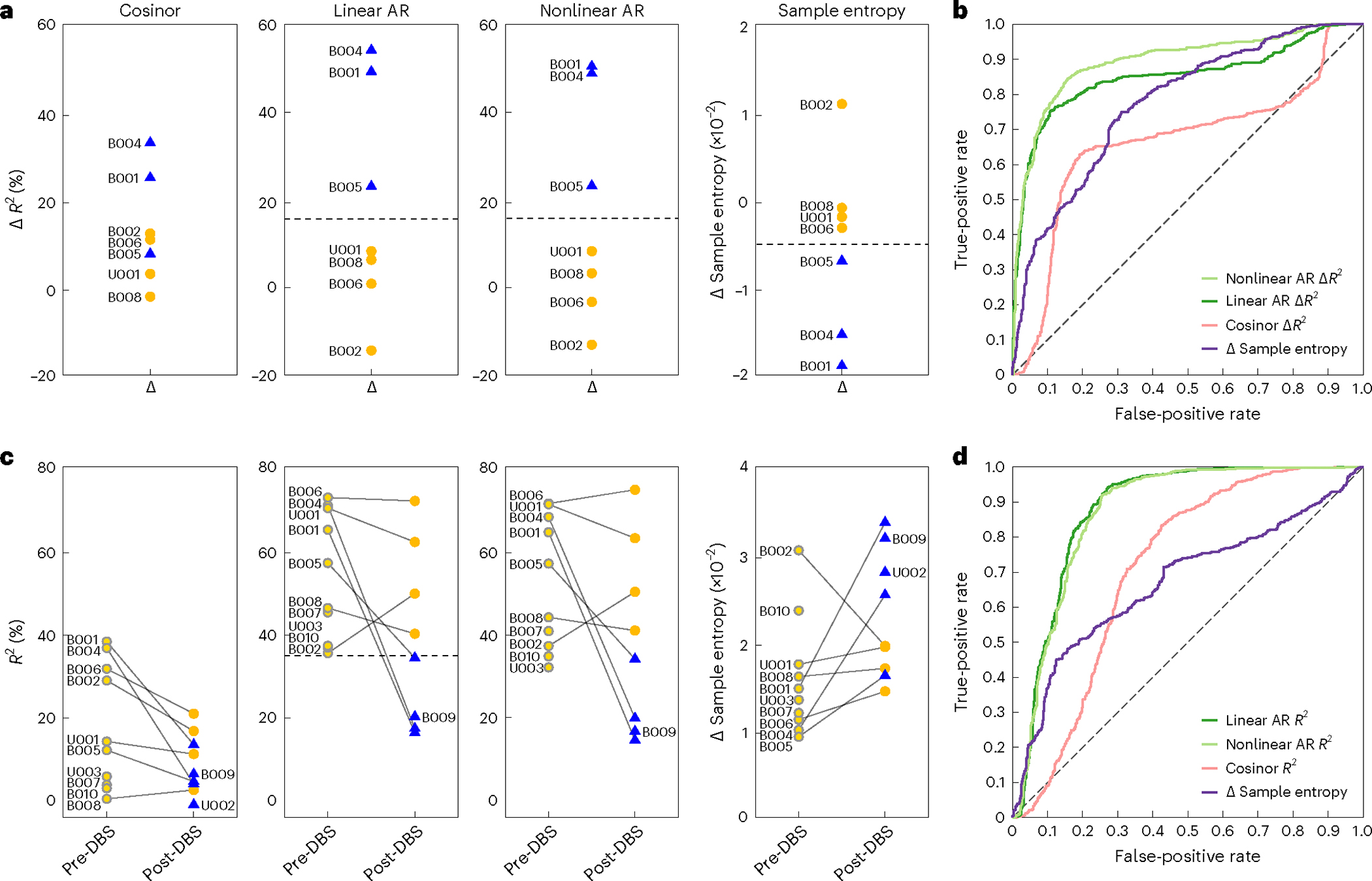
Autoregressive model output metrics accurately distinguish responder status. **a**, Clothesline plots show per-patient delta (that is, normalized to pre-DBS) values for each of the four output measures for the seven patients with pre-DBS and post-DBS data, such that a mean difference could be calculated and five-fold cross-validated (from left to right: cosinor $\Delta R^{2}$, linear autoregressive $\Delta R^{2}$, nonlinear autoregressive $\Delta R^{2}$ and Δ Sample entropy). Blue triangles represent responders, and dark yellow circles represent non-responders. Separation between responders and non-responders was achieved for linear and nonlinear autoregressive $\Delta R^{2}$ values and Δ Sample entropy using a maximum margin classifier (horizontal dashed black line). **b**, ROC curves demonstrate classifier performance (symptom burdened versus symptom unburdened) of a logistic regression model trained and tested on delta values for each of the four output measures (cosinor $\Delta R^{2}$, pink; linear autoregressive $\Delta R^{2}$, green; nonlinear autoregressive $\Delta R^{2}$, light green; and Δ Sample entropy, purple). **c**, Analogous clothesline plots to **a** but including data from all 12 patients, including those with only segments of pre-DBS or post-DBS recordings. U002 does not appear in linear and nonlinear autoregressive $R^{2}$ estimations due to missing data preventing calculation of the daily output measures. Pre-DBS (light yellow circles with gray outlines) and post-DBS non-responder (dark yellow circles) points represent symptom burdened states, and post-DBS responder (blue triangles) points represent symptom unburdened states. Only the linear autoregressive model narrowly achieved separation between symptom burdened and unburdened states. **d**, Analogous ROC curves to **b** but including mean estimates of daily output measures rather than delta values. AR, autoregressive.

We first performed the analyses using data from the seven patients (all five from Cohort 1 and two from Cohort 2) who had both pre-DBS and post-DBS neural data, such that a delta value could be calculated (Fig. 5a; $\Delta R^{2}$ for the autoregressive models and Δ *Sample Entropy* for the model-free measure). For both autoregressive models and sample entropy, a maximum margin classifier could identify a delta value that separated responders from non-responders. The cosinor model was unable to make this distinction.

We then used daily delta values to train a logistic regression model with leave-one-patient-out cross-validation to predict responder status on any given day. We found that linear and nonlinear autoregressive $R^{2}$ measures outperformed cosinor $R^{2}$ and sample entropy measures (Fig. 5b and Supplementary Table 2) and that left hemisphere data yielded more accurate predictions than did right hemisphere data. Left hemisphere linear and nonlinear autoregressive features yielded a balanced accuracy of 82% and 84%, respectively, corresponding to an area under the receiver operating characteristic curve (AUROC) of 85% and 89%, respectively (Extended Data Table 7). Classifier performance was significantly above chance levels (Extended Data Table 7).

An even more demanding requirement would be for a para meter to reliably indicate clinical status based on partial data—that is, recordings lacking a pre-DBS versus post-DBS comparison. Figure 5c includes data from all 12 patients, including those with only a segment of pre-DBS or post-DBS data. Even with these limited data, one of the metrics (the linear autoregressive model) has a maximum margin $R^{2}$ classifier value that separates symptom burdened from unburdened states. The margin between these distributions is very narrow and would likely not hold across a much larger dataset, but these results suggest that the autoregressive model $R^{2}$ value is a strong candidate predictor of clinical status.

As above, we trained a classifier using these daily predictability measures without normalizing to the pre-DBS value. Again, the linear and nonlinear autoregressive $R^{2}$ measures outperformed cosinor $R^{2}$ and sample entropy (Fig. 5d and Supplementary Table 2) and enabled accurate classification of clinical status with a balanced accuracy of 82% and 80%, respectively, corresponding to an AUROC of 88% and 87% (Extended Data Table 7). This performance was, again, well above chance (Extended Data Table 7). Thus, despite the lack of pre-DBS and post-DBS recordings in the expanded cohort of 12 patients, predictability measures from the autoregressive model fits were still able to reliably classify responder status.

## Discussion

Our results identify a neurophysiological biomarker in the VS that enables prediction of clinical state from neural data. The best-performing feature—daily predictability of low-frequency (~9 Hz) power—is most prominent in the severely symptomatic state. Chronically, retention versus loss of this predictability distinguishes clinical non-responders from responders, respectively. We initially identified this neurobehavioral relationship in a cohort of five patients with plentiful data, and its robustness was maintained in an expanded cohort of seven additional patients with sparser data.

A consistent relationship between a readily trackable neural feature and clinical status would have important practical implications. Such a biomarker could help determine whether an individual is heading in the direction of clinical response. Programming DBS for OCD is challenging because of the inconsistent relationship between DBS parameter adjustments and symptomatic changes. Unlike the field of movement disorder DBS, in which adjustments are often associated with immediate changes in symptoms (for example, reduction in tremor or stiffness), reduction in OCD symptom severity per se often requires months to manifest. An early neural indicator of emerging treatment response would be extremely informative and could help guide therapy delivery, thus demystifying the process of DBS programming for OCD and making the therapy more accessible to a greater number of clinicians and patients^39,40^.

Further contributing to the generalizability of our findings is the straightforward nature of obtaining these neural recordings. The presence of the spectral feature that we observed in the first few participants near the theta/alpha border was consistent with the relevance of this frequency range to the cognitive processes underlying OCD^27,28^. The prominence and exact center frequency of this approximately 9-Hz feature varied across the 12 patients, but our results demonstrated robustness even with all patients’ recordings fixed at 9 Hz. Avoiding the need to individualize recording settings reduces complexity and increases the likelihood that novice centers adopt and make use of these findings. Data availability and utility are also facilitated by the passive nature of the recordings. Our previous work identified low-frequency neural correlates of OCD symptom severity^20^, but the active data acquisition requirement demanded considerable effort from the research team as well as exceptional commitment from the patients. Reliable results obtained from passive data streams will further democratize their accessibility and usability.

The availability of chronic, continuous neural data provides new perspectives for neurobehavioral analyses. Traditionally, neural biomarkers of psychiatric symptom states have relied on snapshots of neural data collected during behavioral tasks, time-locked exposures to triggers or variation in symptom severity. Such measures may be appropriate in the context of adaptive or responsive neuromodulation development, which prioritizes relatively rapid adjustments of stimulation delivery based on episodic changes in symptom state. Our findings, on the other hand, indicate that neural biomarkers of slowly evolving clinical states may not be episodic variations from baseline but, rather, features of variation in the baseline itself. These high-amplitude periodic variations must be taken into account when studying the paroxysmal changes, as the simple effect of time of day will otherwise obscure interpretability. Failure to do so may still produce statistically significant and meaningful relationships between clinical state and neural activity but would fail to fully capture the dynamics.

These results have a direct implication on efforts related to closed-loop neuromodulation therapy. DBS adjustments (and even medication administration) in Parkinson’s disease, for example, have rapid effects on motor symptoms. OCD, on the other hand, is an example of a disorder in which symptom changes usually take weeks to months after DBS initiation or adjustment to become evident. Consistently, the principal tool used to measure OCD symptom severity, the Y-BOCS, asks the patient to consider an approximately week-long period in their responses. Applying this concept to the design of closed-loop algorithms, the temporally tight neural symptom relationship in Parkinson’s disease has led to the use of very short time constants relating detection of a neural signal (for example, increased beta power in the subthalamic nucleus) and adjustment of DBS settings^41^. The slower neurobehavioral time constant in our results, akin to the one for depression severity recently described by others^42^, suggests that building closed-loop classifiers for psychiatric disorders may benefit from using slowly evolving neural signals (for example, daily measures of neural predictability) rather than episodic ones.

An important question raised by these results is that of a mechanistic explanation: what neurophysiological processes underlie the circadian periodicity and neural predictability that we observed that seem so closely related to clinical state? Although our data cannot answer this question, the literature suggests physiological and anatomical relationships that may be explanatory and could generate testable hypotheses for future animal or human research.

One possibility is that the 9-Hz circadian rhythm that we observed is produced by pattern generators in the hypothalamus, as are many other such rhythms^43^. Among the most common cell types related to circadian pattern generation are the orexin neurons of the lateral hypothalamus, which regulate not only wakefulness and arousal but also limbic processes, including fear, stress response and anxiety^44,45^. Their pathological activity may induce hypervigilant states that promote fearful/avoidant behavior characteristic of the OCD symptomatic state^44,45^. From the anatomical point of view, the DBS target borders the VS but may, in addition, impinge on the gray matter of the bed nucleus stria terminalis (BNST), a small paired septal nucleus that, in animals, is involved in fear conditioning^46^. Due to the direct connectivity between VS/BNST and the hypothalamus, DBS in this region may have direct physiological effects on the hypothalamus^47^. Combining these lines of evidence, it is possible that pathological activity of pattern generators, such as orexin neurons, produces the avoidant behaviors observed in OCD concomitantly with the strongly periodic neural activity^44,45,48^. DBS of the VS/BNST region may interfere with this lateral hypothalamic dysfunctional activity, disrupting the neural pattern and reversing behavior toward a more approachful diathesis characterized by tolerance of previous triggers and, thus, decreased OCD symptom severity.

These hypothalamic effects may help explain the daily periodicity that we observed in neural activity in the symptomatic state (for example, Fig. 3c). Atop this 24-h cycle was a higher-frequency neural activity pattern with a high degree of predictability before DBS that persisted after DBS in non-responders. This neural predictability is consistent with the predictability in behavior that characterizes the OCD symptomatic state. Individuals with OCD are often seized by rituals, whether overt (for example, washing, checking, touching, counting and repeating) or covert (for example, silently reciting prayers to neutralize unwanted thoughts). The need to perform these rituals defines these individuals’ response to a situation or trigger and, thereby, severely limits their behavioral repertoire, making it highly predictable when exposed to triggers. This rigid repertoire and perseverative behavior may stem from the cognitive inflexibility that has long been postulated to underlie the OC phenotype^9^.

In contrast, healthy, adaptive behavior is characterized by the flexibility to respond to external or internal cues in the context of longer-term goals and plans. Indeed, cognitive flexibility is one of the hallmark features of intelligent behavior^49^. The transition from repetitive, ritualistic actions in the symptomatic state to more adaptive and flexible behaviors in the responder state is accompanied by increased dispersion and decreased predictability of VS neural activity. Our working hypothesis is that greater variability in neural activity in the VS, a structure known to influence motivated, goal-directed behavior^50^, allows the agent to shed maladaptive ritualistic behavior in favor of reasoned behavior aligned with long-term goals.

An immediate goal of our future work is to improve the granularity and objectivity of behavioral quantification outside the clinical setting. Our neural data are continuously available, but our behavioral/clinical data are relatively sparse and limited to our direct observations or to caregiver reports. Behavioral data with greater temporal resolution would allow us to study in detail the neurobehavioral trajectory from before DBS activation to the clinical plateau. Pairing the dense, passive neural recordings with equally dense and passive (and, therefore, feasibly obtainable) behavioral data from time-synchronized peripheral/wearable devices would make great strides toward this goal.

This transition toward dense, naturalistic, passively acquired neurobehavioral data would take research on OCD and other disorders directly to the lived lives of affected individuals. Doing so would not only increase the relevance of the results to patients but also facilitate data acquisition and therapy management, thus enabling more research groups to further explore these neurobehavioral relationships. This feed-forward system would hopefully shed greater light on the neurophysiology of psychiatric disorders, better contextualize them within the cognitive neuroscience framework^8^ and ultimately improve outcomes^14^ and access^39,40^ to these therapies for the tremendous number of affected individuals.

### Online content

Any methods, additional references, Nature Portfolio reporting summaries, source data, extended data, supplementary information, acknowledgements, peer review information; details of author contributions and competing interests; and statements of data and code availability are available at https://doi.org/10.1038/s41591-024-03125-0.

## Methods

### Study design

Twelve adults with a principal diagnosis of severe, treatment-resistant OCD underwent DBS implantation after informed consent (NCT05915741). All procedures were approved by the local institutional review board (IRB) at Baylor College of Medicine (BCM; IRB H-48392) and the University of Utah (IRB_00169174). Patients were not compensated for any of the research described here. All patients had OCD for more than 5 years and had failed adequate trials of selective serotonin reuptake inhibitors (SSRIs), clomipramine and augmentation as well as expert ERP therapy. Patient demographics are included in Table 1. Nine patients (B001–B010) underwent DBS surgery and were followed at BCM, and three patients underwent DBS surgery and were followed at the University of Utah (U001–U003).

The full group consisted of two patient cohorts. Cohort 1 consisted of the first five patients (B001, B003, B004, B005 and B006) from whom we have the longest recordings, starting before DBS initiation and spanning months afterwards until they achieved stable chronic clinical status. Data from these patients were used to construct the neurobehavioral models. Cohort 2 consisted of the next seven patients (B007–U003) whose data are more limited for several reasons. One of these patients (B009) received the recording-enabled DBS generator at the time of a generator replacement procedure, such that recordings were only available well into their therapy. Three patients (B007, U001 and U002) underwent a programming change soon after DBS initiation to a recording-incompatible stimulation configuration, precluding recordings for a duration of their therapy. Three patients (B008, B010 and U003) were implanted relatively recently or have shorter clinical follow-up (less than 6 months) and, therefore, do not have recordings spanning the full trajectory from pre-DBS to stable chronic status.

Gender (as reported in Table 1) was determined based on self-report. Neither sex nor gender was considered in the study design, and sex/gender analyses were not carried out due to the small sample size and lack of pre-existing hypotheses on sex/gender differences.

### DBS surgery

DBS leads (model 3387 or SenSight) with 1.5-mm spacing were placed bilaterally in the VC/VS region. Lead locations were determined using direct targeting on the pre-operative magnetic resonance imaging (MRI). We refined the location of the leads using awake intra-operative testing as we previously described^19^. The pair of leads was connected to extensions that were tunneled to a Medtronic Percept PC pulse generator.

### Imaging protocol and DBS electrode localization

We acquired pre-operative high-resolution T1-weighted MRI sequences and post-operative computed tomography (CT) as previously described^20^. Electrode reconstruction was performed using the advanced processing pipeline in Lead-DBS^51^ (version 2.5; https://www.lead-dbs.org/). Lead Group software was used to visualize all electrodes from single-implant and dual-implant patients using the DISTAL Minimal Atlas in 7T MRI ex vivo atlas^52–54^ (Fig. 1a).

### Determination of clinical states

The Y-BOCS (ref. 55) and the Y-BOCS II (ref. 56) were administered before surgery and subsequently periodically throughout the course of DBS treatment. We supplemented these clinical indicators with reports obtained from reliable informants (for example, caregivers and family members) whose communications we considered trustworthy. This combination of data takes advantage of impressions from both clinicians and caregivers and reflects the real-world nature of this study. We used these data to classify four clinical states. (1) Severe OCD symptoms. By definition, this was the state before DBS initiation. (2) Clinical response. We classified clinical response as periods when a patient demonstrated clinically meaningful improvement in OCD symptoms, defined quantitatively as ≥35% improvement in Y-BOCS (conventional ‘responder status’) or qualitatively as significant improvement allowing successful return to professional/social life. (3) Persistent OCD symptoms. These were periods after DBS initiation during which OCD symptom severity remained near pre-DBS levels. (4) Disinhibited behavior. We defined these as periods when the patient exhibited disinhibition (recklessness, increased libido and poor decision-making) to an extent that warranted DBS adjustment. Unlabeled periods are those during which clinical status is undetermined (that is, insufficient data) or intermediate (that is, improved but not reaching clinical response criteria).

### Time-domain neural recordings

Time-domain neural recordings were acquired directly from the Percept PC device at a sample rate of 250 Hz. During recordings with DBS on, we used BrainSense Streaming mode to record LFPs in a bipolar configuration around the active stimulation contact. During recordings with DBS off, we leveraged Indefinite Streaming mode to enable recordings from each of the three possible contact pairs (0–2, 1–3 and 0–3; assuming monopolar stimulation at contact 1 or 2) simultaneously.

### Power spectral density estimations

To analyze the oscillatory content of the time-domain LFP data, we estimated power spectral density from data collected while the patient was at rest. First, we computed power spectral density for the time-domain signal using Welch’s method (MATLAB pwelch function) with a Hamming window of 1 s, a window overlap of 600 ms and a 256-point fast Fourier transform (FFT). We then converted to decibels by taking the base 10 logarithm of the result and multiplying by 10. The resulting power spectral density plots (power in decibels versus frequency) are shown in Fig. 1.

### Continuous spectral amplitude recordings

#### Data collection.

The Percept PC can be configured to record a continuous estimate of average LFP amplitude from a user-defined frequency band of interest (center frequency ±2.5 Hz). These data are saved onboard the device every 10 min (144 samples per day) and can be offloaded upon connection to the Medtronic DBS Clinician Programmer Application. We configured the device to record LFP signal amplitude (units of microvolts peak) in the 8.79 ± 2.5 Hz band (referred to in the main text and hereafter as 9 Hz).

#### Data downloads.

These chronic data were exported in JSON format during clinic visits. Gaps in data occurred for one of two reasons: (1) errors in recording configuration or (2) if more than 60 d have passed since the data were last downloaded. After 60 d, the device begins to overwrite already saved data due to onboard storage limitations.

### Data pre-processing

#### Data conditioning.

Timestamps were converted from UTC to local time zone. Outliers were defined as values above 30 standard deviations over the global median of a continuous chunk of data with no losses and were then replaced with a piecewise cubic hermite interpolating polynomial (pchip) method. Less than 0.1% of data met the criteria for outliers. Subsequently, segments of missing values with a length of, at most, 1 h (six samples) were interpolated. Otherwise, missing timepoints were stored as not a number (NaN). Interpolation was used for less than 0.17% of data, and the average interpolation length was 1.78 ± 1.28 samples.

#### Normalization.

To account for increases over time in baseline LFP amplitude and stimulation amplitude, daily power arrays were *z*-scored using daily means and standard deviations.

#### Visualization.

The raw amplitude values were discretized into one of 144 10-min bins based on timestamp and reshaped from a one-dimensional vector of chronological data to a two-dimensional matrix of date versus time of day for visualization. These data were then plotted as a heatmap (Fig. 2) where greater power values are represented by lighter blue colors.

### Construction of circularized plots of daily 9-Hz power

To better visualize the 9-Hz power fluctuations over the course of the 24-h period, we generated circularized polar plots of 9-Hz power on each day.

#### Rotation and smoothing.

To reduce the impact of slow phase shifts on data visualizations and averaging (see below), the polar plots were constructed such that data from each 24-h period were rotated to align peak activity to midday (12:00 local time). For each day in which an acrophase was fit with a significant *P* value, the power values for that day were rotated circularly so that the acrophase pointed to 3π/2 radians (downward on the polar plot). A Gaussian smoothing blur kernel was applied to each day after rotation.

#### Visualizations.

Representative examples of the pre-DBS severe OCD symptom (light yellow), clinical response (blue) and persistent OCD symptom (dark yellow) are shown in Figs. 3 and 4. Single-day circularized plots were visualized over time in Supplemental Videos 1 and 2 for patients B004 and B006.

### Overview of model-based and model-free measures

To analyze the periodicity and predictability of our data, we applied a suite of model-based measures (cosinor $R^{2}$ and linear and nonlinear autoregressive $R^{2}$) and one model-free measure (sample entropy). For each of these four output measures using data collected in the VS, we computed an average representing the pre-DBS severe OCD symptom state, the post-DBS persistent OCD symptom state and the post-DBS clinical response state. In addition to the average measures computed per state, we computed these measures on each day so that we could visualize the trajectory over time and build distributions that are representative of each clinical state. The following sections describe the metrics and the respective analyses related to each in more detail.

### Cosinor analysis and statistics

#### Cosinor model.

Normalized LFP data were fitted to a sum of cosine functions using a linear regression model (equation (1)), as described by Moškon et al. (statsmodel 0.14.0)^57^. The fitted sinusoid with *N* components can be expressed as:

(1) $$y\left(t\right)=M+\sum_{j=1}^{N}A_{j,1}sin\left(\frac{2\pi jt}{P}\right)+A_{j,2}\times cos\left(\frac{2\pi jt}{P}\right)+e\left(t\right)$$

Here, y(t) represents the raw signal; e(t) is an error term; M is the midline statistic of rhythm (MESOR); P is the period of the cosinor used to fit the data; A is amplitude; j is component; and t is time. A period of exactly *P* = 24 h was selected for all analyses. The number of sinusoidal components was selected per patient to reflect the maximum number of daily peaks.

Cosine fits were generated using 5-d windows centered on the day of interest (for example, day of interest ±2 d). To determine strength of periodicity before DBS, we also fit a single cosinor using the pre-DBS severe symptom state for each patient (Supplementary Table 1).

#### Cosinor metrics.

Cosine fits were expressed as acrophase (for example, time shift of daily cosine peak from 0:00 local time), amplitude and the coefficient of determination ($R^{2}$). The resulting phase and amplitude values are plotted per patient in the polar plots shown in Fig. 2 and Extended Data Fig. 1. Amplitudes and acrophases reported here display only the ‘primary’ (that is, largest) peak.

The cosinor model was fitted to individual patient states (that is, pre-DBS, persistent OCD symptoms and clinical response) using a five-fold cross-validation strategy implemented across all 10-min timepoints. For each fold, we used the model to generate a prediction of the *z*-scored data at each 10-min timepoint and calculated an $R^{2}$ value representing the strength of the fit. These $R^{2}$ values were then averaged across all test folds within each clinical state to generate an average $R^{2}$ value representative of each clinical state, per patient. We then aggregated the predicted data points from all five models to calculate an $R^{2}$ value representing the strength of the fit on each day. The cross validated $R^{2}$ values and daily $R^{2}$ values (equation (2)) were calculated using the scikit-learn 1.2.2 Python package and can be expressed as:

(2) $$R^{2}=1-\frac{\sum_{i=1}^{n}\;{\left(y_{i}-{\overset{ˆ}{y}}_{i}\right)}^{2}}{\sum_{i=1}^{n}\;{\left(y_{i}-\overset{‾}{y}\right)}^{2}}$$

where $y_{i}$ is the true value; ${\overset{ˆ}{y}}_{i}$ is the predicted value at sample i; and $\overset{‾}{y}$ is the mean of true values.

### Linear autoregressive model analysis and statistics

A linear autoregressive model is a statistical model used for predicting time-series data, where the current value in a series is modeled as a linear combination of previous values. A linear autoregressive model (equation (3)) can be expressed as:

(3) $$X_{t}=c+\Phi_{1}X_{t-1}+\Phi_{2}X_{t-2}+\cdots +\Phi_{p}X_{t-p}+\varepsilon_{t}$$

Here, $X_{t}$ is the value of the series at time t;c is a constant; $\phi_{k}$ are p parameters of the model; and $\varepsilon_{t}$ is an error term.

#### Determining significant lag terms.

Lag terms including up to 144 previous timepoints (from $X_{t-1}$ to $X_{t-144}$) were used in the model to predict the value of the series at each time, t. To prevent overfitting, we selected significant lag terms from the time-series data for each patient. The model initially considered one day’s worth of previous values, amounting to 144 timepoints, as potential regressors. To determine the robustness and relevance of these lag terms, a five-fold cross-validation technique was conducted with an ordinary least squares regression model. During each fold of the cross-validation, lag terms that showed statistical significance (*P* < 0.05) were identified and recorded. A lag term was retained for further analysis if it was deemed significant in more than three of the cross-validation folds. This process ensured that only consistently relevant lag terms were considered and prevented overfitting of the model. Subsequently, all selected significant lag terms were tested against the entire dataset. In cases where any lag terms proved to be statistically non-significant when applied to the full dataset, the above selection process was iteratively repeated. The process continued until only statistically significant lag terms remained.

#### Training and evaluation.

After identifying a set of statistically significant lag terms for each patient’s entire dataset, we applied a linear autoregressive model to individual patient states (that is, pre-DBS, persistent OCD symptoms and clinical response). To assess the model’s predictive accuracy within each state, we employed a five-fold cross-validation strategy implemented across all 10-min timepoints. The key metric for evaluating the model’s performance was the coefficient of determination ($R^{2}$). Within each fold, we used the model to generate predictions of the data at each 10-min timepoint. Similar to the cosinor model evaluation, we aggregated datapoints across all five models to calculate an $R^{2}$ value representing the strength of the fit on each day. The results of our analysis were robust to heteroskedastic conditions, as confirmed by implementing a heteroskedastic autocorrelation robust covariance in our regression, which yielded predictions consistent with those from a standard regression.

### Nonlinear autoregressive model analysis and statistics

A nonlinear autoregressive model extends the scope of a linear autoregressive model by capturing complex, nonlinear relationships within the data. We modeled the relationship between past data points and current values using a neural network due to the expressive nature of these models for nonlinear relationships.

#### Neural network architecture.

We designed a simple neural network architecture using PyTorch 2.1.0+cu118. The network is composed of two layers. The initial layer of the network is a fully connected linear layer that transforms the input, consisting of 144 lagged features of the previous day, into a hidden layer comprising 32 neurons. A nonlinear activation function, rectified linear unit (ReLU), is applied after the first layer to introduce nonlinearity into the model. The second layer then maps the 32-neuron hidden layer to the single-neuron output layer that predicts the time series.

#### Training and evaluation.

Similar to the linear autoregressive model, we applied the nonlinear autoregressive model to individual patient states (that is, pre-DBS, persistent OCD symptoms and clinical response). Within each state, a five-fold cross-validation strategy was implemented across all 10-min timepoints. The training for each cross-validation split was conducted over 50 epochs, using the Adam optimizer with a learning rate of 0.001 to optimize performance. To prevent overfitting and to enhance the model’s generalizability, L1 regularization was applied with a lambda of 10^−5^. The mean absolute error served as the loss function. The model architecture, the loss function, the regularization scheme and the learning rate were all optimized through testing. Throughout the training phase, we monitored the $R^{2}$ for both training and testing sets as a function of epochs to prevent overfitting. We selected the epoch with the best test $R^{2}$ as our best model for each fold in our cross-validation strategy. Similar to the cosinor and linear autoregressive model evaluation strategies, we aggregated data points from all five models to calculate an $R^{2}$ value measuring the goodness of the fit on each day.

### Sample entropy analysis and statistics

Sample entropy is a model-free measure used to quantify the regularity and (un)predictability of time-series data^58^. Intuitively, this measure provides an estimate of the complexity in a time-series by quantifying the similarity of small windows within the time-series.

Mathematically, sample entropy (equation (4)) is defined and calculated as:

(4) $$\text{Sample}\;\text{entropy}=-\ln\left(\frac{A}{B}\right),$$

where A is the number of unique pairs of distinct subsequences/windows $\left(x_{i},x_{j}\right)$ of X of length m+1, and B is the number of unique pairs of distinct subsequences/windows $\left(x_{i},x_{j}\right)$ of X of length m. The Manhattan distance $d\left(x_{i},x_{j}\right)$ between any two distinct subsequences/windows must be smaller than a user-specified tolerance, r, to be considered similar. The implementation of sample entropy was based on the EntropyHub version 0.2 package^59^. To generate sample entropy values for each day, we used single-day *z*-scored 9-Hz power values as input and set the input parameters as m=2,r=3.6 and τ=1.

### Quantification of chronic changes in neural activity in response to VS stimulation

#### Statistical calculations.

To compare the mean difference between the pre-DBS state (light yellow) and post-DBS clinical response state (blue) or the persistent OCD symptom state (dark yellow), we computed a delta value representing the difference between the pre-DBS and post-DBS mean values for each of the four output measures described above (cosinor $R^{2}$, linear autoregressive $R^{2}$, nonlinear autoregressive $R^{2}$ and sample entropy) (Fig. 5a).

We quantified the separation between symptom burdened (for example, pre-DBS and persistent OCD symptoms) and symptom unburdened (for example, clinical response) states by using a maximum margin classifier to demonstrate the linear separability of average output measures across these two states. The maximum margin classifier is visualized as a horizontal dotted line in Fig. 5a,c for the average deltas and average daily output measures, respectively.

Within each patient, the four daily output measures described above were compared between the pre-DBS and the post-DBS distributions using two-sample two-tailed Welch’s *t*-tests. We report the results of the *t-*test and modified *t-*test both before and after sample size correction, respectively.

To account for the autocorrelation present within each distribution, we computed a measure of true information present in the data known as effective sample size (ESS) (equation (5)). ESS quantifies the number of independent and identically distributed samples that would be equivalent to the correlated samples in terms of the amount of information that they contain about the population parameter. The ESS of highly autocorrelated data is much smaller than the actual sample size because correlated samples contain less unique information. Mathematically effective sample size is expressed as:

(5) $$\text{Effective}\;\text{sample}\;\text{size}=\frac{N}{1\;+\;2\sum_{k=1}^{m}\;p_{k}},$$

where N is the original sample size, and $p_{k}$ is the autocorrelation coefficients at lag k up to a maximum lag m. In our analysis, we chose a maximum lag of 10 d. For distributions with fewer than 10 d of data, the maximum lag was the number of days present in the distribution. The effective sample size was then used to calculate a two-sample two-tailed Welch’s t-test and a Hedge’s g in place of a traditional sample size. Results of these statistical analyses are included in Extended Data Tables 1–5 for both left and right VS.

Lastly, we then pooled the data across patients and computed two-sample two-tailed Welch’s t-tests comparing the daily output measures associated with symptom burdened and symptom unburdened states. The effective sample size for each output measure was calculated as the sum of the effective sample size of each individual patient’s data included in the analysis. Results of both the corrected and uncorrected tests are included in Extended Data Table 6 for each of the four output measures for both left and right VS.

### Logistic regression binary classifier construction

#### Classifier development.

We used a logistic regression model to predict clinical status (symptom burdened versus symptom unburdened) on any given day. We trained the model a total of eight times on different sets of input features. The first four classifiers were trained using the ‘delta values’ corresponding to each of the four output measures, where the delta value corresponds to the difference between the average output measure for the pre-DBS state and the output measure on any given day. These features can be calculated only if both pre-DBS and post-DBS data are available within the same patient. The second set of four classifiers was trained without the pre-DBS and post-DBS comparison and, instead, used the raw output measures calculated on each day as feature inputs. Therefore, these features can be calculated for any patient regardless of missing data.

We balanced the class weights of the logistic regression model proportionally to reflect the class imbalance in the data for each patient and performed leave-one-patient-out cross-validation to test the model. This process was repeated until data from all patients were tested. We computed the ROC curve, the AUROC and the balanced accuracy for all the classifiers (equation (6)) (Extended Data Table 7).

(6) $$\text{Balanced}\;\text{accuracy}\;=\frac{\text{Sensitivity}\;+\;\text{Specificity}}{2}\;\;=\frac{1}{2}\left(\frac{\text{TP}}{\text{TP}\;+\;\text{FN}}+\frac{\text{TN}}{\text{TN}\;+\;\text{FP}}\right)$$

where TN,TP,FN and FP denote true negative, true positive, false negative and false positive rates, respectively.

#### Chance performance estimation.

Due to the imbalance of data between the two states (that is, non-responder versus responder), we performed a permutation test to determine chance level performance of the classifier and whether classifier performance was significantly different than chance. We assigned labels in two different ways: (1) randomly and (2) via a circular shift.

For the first permutation test, the labels between the two states were randomly shuffled. For the second permutation test, the labels for each patient were circularly shifted by a random amount. We implemented this shuffling strategy to preserve the temporally contiguous nature of the data labels (that is, response/non-response labels do not occur randomly but, rather, in large contiguous blocks). A random shuffling procedure would reduce the amount of autocorrelation between the train/test splits and, thereby, inflate the difference between the true value and shuffle distribution.

For both methods, we then used a leave-one-patient-out cross-validation strategy (as previously described) to evaluate the model’s performance on the shuffled data. As before, we computed the AUROC score and balanced accuracy to quantify performance. This procedure was repeated 10,000 times to create a chance classification distribution for each patient. The results are tabulated in Extended Data Table 7.

#### Statistical significance testing.

To determine if the classifier performance using true labels significantly outperformed chance performance, we compared the true test metrics (AUROC score and balanced accuracy measures) with the chance distributions estimated for each classifier. To estimate *P* values, we implemented a randomization test where we counted the number of values in the distribution that were greater than the test metric and divided the result by the total number of permutations (10,000). The results are reported in Extended Data Table 7.

#### Model comparison.

Within each model (that is, delta or daily model), the classifier performance was compared across the four different sets of feature inputs (that is, output measures) in a pairwise fashion using a non-parametric statistical significance test (DeLongʼs test). This test allows for comparison of two AUROCs calculated on the same dataset. The analysis was performed using the auroc-matlab package in MATLAB (https://github.com/alistairewj/auroc-matlab). Results of the DeLongʼs test are included in Supplementary Table 2.

## Extended Data

**Extended Data Table 1 |:** Cosinor model fit of 9-Hz VS power pre-DBS versus post-DBS in Cohort 1 patients

		Cosinor $R^{2}$ Left Hemisphere					Cosinor $R^{2}$ Right Hemisphere				
	
		B001	B002	B004	B005	B006	B001	B002	B004	B005	B006

**Uncorrected Sample Size**	**P-value**	1.02e-16	0.264	4.17e-5	3.33e-3	0.066	2.92e-3	0.314	--	1.49e-6	1.6e-6
	
	**T-statistic**	−10.4	−1.25	−7.88	−3.08	−2.00	−3.09	−1.12	--	−5.54	−8.66
	
	**95% CI**	[−0.304, −0.207]	[−0.314, 0.107]	[−0.398, −0.218]	[−0.118, −0.025]	[−0.208, 0.008]	[−0.101, −0.022]	[−0.376, 0.147]	--	[−0.184, −0.086]	[−0.484, −0.29]
	
	**DF**	83.2	5.26	8.23	50.4	13.7	67.5	5.1	--	45.6	12
	
	**Hedges G**	−2.07	−0.579	−2.92	−0.563	−0.486	−0.618	−0.517	--	−1.1	−3.11
	
	**Hedges G CI**	[−2.57, −1.57]	[−1.51, 0.384]	[−4.10, −1.71]	[−0.928, −0.194]	[−0.966, −0.002]	[−1.02, −0.210]	[−1.44, 0.437]	--	[−1.53, −0.657]	[−4.45, −1.74]
	
	**N_1_**	48	6	9	44	13	48	6	9	44	13
	
	**N_2_**	53	74	317	239	323	53	182	0	39	413

**Corrected (Effective) Sample Size**	**P-value**	1.19e-6	0.690	0.136	0.123	0.435	0.157	0.631	--	0.016	0.028
	
	**T-statistic**	−6.36	−0.637	−3.84	−1.63	−0.964	−1.51	−0.647	--	−2.77	−4.53
	
	**95% CI**	[−0.338, −0.173]	[−12.6, 12.4]	[−1.05, 0.438]	[−0.164, 0.022]	[−0.540, 0.340]	[−0.150, 0.027]	[−2.16, 1.93]	--	[−0.240, −0.030]	[−0.686, −0.088]
	
	**DF**	24.93	0.583	1.15	15.9	2.04	11.7	1.04	--	12.9	2.57
	
	**Hedges G**	−2.14	−0.859	−3.68	−0.622	−0.434	−0.696	−0.819	--	−1.20	−8.09
	
	**Hedges G CI**	[−2.93, −1.34]	[−2.61, 0.891]	[−5.13, −2.24]	[−1.28, 0.033]	[−1.60, 0.732]	[−1.47, 0.086]	[−2.27, 0.629]	--	[−2.02, −0.378]	[−9.63, −6.55]
	
	**N_eff 1_**	16	2	2	13	3	10	2	2	12	4
	
	**N_eff 2_**	25	21	117	39	165	23	52	0	19	101

Two-sample two-tailed Welch’s *t*-tests (calculated using either the uncorrected sample size (top half of table) or the effective sample size (bottom half)) were conducted for the pre-DBS versus post-DBS periods using parameters of cosinor coefficient of determination ($R^{2}$). For the pre-DBS and post-DBS states, *N_1_* and *N_2_* denote the uncorrected sample sizes, and *N_eff1_* and *N_eff2_* denote the effective sample sizes. Results for the right hemisphere of patient B004 are not available because of a recording-incompatible configuration. Clinical responders are highlighted in light blue. CI, confidence interval; DF, degrees of freedom.

**Extended Data Table 2 |:** Linear autoregressive model fit of 9-Hz VS power pre-DBS versus post-DBS in Cohort 1 patients

		Linear AR $R^{2}$ Left Hemisphere					Linear AR $R^{2}$ Right Hemisphere				
	
		B001	B002	B004	B005	B006	B001	B002	B004	B005	B006

**Uncorrected Sample Size**	**P-value**	2.46e-40	0.142	3.40e-9	1.11e-9	0.51	1.32e-22	0.733	--	4.58e-47	1.77e-32
	
	**T-statistic**	−24.5	1.7	−18.6	−7.32	−0.676	−13.1	−0.359	--	−21.2	−30.7
	
	**95% CI**	[−0.538, −0.458]	[−0.073, 0.398]	[−0.592, −0.466]	[−0.276, −0.157]	[−0.043, 0.023]	[−0.408, −0.301]	[−0.205, 0.154]	--	[−0.431, −0.357]	[−0.523, −0.458]
	
	**DF**	85	5.84	10.2	55.3	14.3	89.4	5.44	--	151	46.4
	
	**Hedges G**	−4.78	0.691	−3.93	−1.25	−0.151	−2.61	−0.135	--	−2.49	−2.97
	
	**Hedges G CI**	[−5.60, −3.96]	[−0.145, 1.51]	[−4.53, −3.33]	[−1.62, −0.879]	[−0.589, 0.288]	[−3.15, −2.07]	[−0.874, 0.606]	--	[−2.8, −2.18]	[−3.24, −2.69]
	
	**N_1_**	48	6	9	44	13	48	6	9	44	13
	
	**N_2_**	53	74	317	239	323	53	182	0	39	413

**Corrected (Effective) Sample Size**	**P-value**	3.42e-17	0.513	6.10e-4	0.004	0.799	5.21e-5	0.886	--	1.15e-8	7.69e-14
	
	**T-statistic**	−15.4	0.961	−10.3	−3.56	−0.298	−5.89	−0.193	--	−9.85	−11.9
	
	**95% CI**	[−0.564, −0.432]	[−2.00, 2.33]	[−0.674, −0.384]	[−0.350, −0.083]	[−0.193, 0.173]	[−0.484, −0.224]	[−3.72, 3.66]	--	[−0.478, −0.310]	[−0.574, −0.406]
	
	**DF**	35.2	0.996	3.84	11.4	1.66	13.1	0.759	--	18	35
	
	**Hedges G**	−4.53	0.702	−3.09	−1.33	−0.128	−2.62	−0.13	--	−1.97	−2.19
	
	**Hedges G CI**	[−5.77, −3.28]	[−0.836, 2.24]	[−4.41, −1.78]	[−2.03, −0.621]	[−1.41, 1.16]	[−3.84, −1.41]	[−1.67, 1.41]	--	[−2.80, −1.15]	[−3.29, −1.09]
	
	**N_eff 1_**	16	2	3	10	2	8	2	2	8	5
	
	**N_eff 2_**	22	32	47	66	88	14	69	0	66	39

Two-sample two-tailed Welch’s t-tests (calculated using either the uncorrected sample size (top half of table) or the effective sample size (bottom half)) were conducted for the pre-DBS versus post-DBS periods using parameters of linear autoregressive coefficient of determination ($R^{2}$). Abbreviations are the same as in Extended Data Table 1.

**Extended Data Table 3 |:** Nonlinear autoregressive model fit of 9-Hz VS power pre-DBS versus post-DBS in Cohort 1 patients

		Nonlinear AR $R^{2}$ Left Hemisphere					Nonlinear AR $R^{2}$ Right Hemisphere				
	
		B001	B002	B004	B005	B006	B001	B002	B004	B005	B006

**Uncorrected Sample Size**	**P-value**	1.78e-45	0.087	3.68e-8	5.05e-9	0.05	4.07e-26	0.819	--	2.49e-43	1.09e-39
	
	**T-statistic**	−27.5	2.01	−14.7	−6.96	2.15	−16	0.24	--	−20.6	−31.6
	
	**95% CI**	[−0.543, −0.469]	[−0.031, 0.350]	[−0.543, −0.400]	[−0.279, −0.154]	[<10^−4^, 0.075]	[−0.471, −0.367]	[−0.138, 0.168]	--	[−0.421, −0.347]	[−0.466, −0.410]
	
	**DF**	89.9	6.54	10.1	53.5	13.7	76.5	5.7	--	134	61.3
	
	**Hedges G**	−5.37	0.727	−3.15	−1.22	0.53	−3.19	0.08	--	−2.5	−2.88
	
	**Hedges G CI**	[−6.25, −4.48]	[−0.009, 1.45]	[−3.69, −2.60]	[−1.59, −0.835]	[0.039, 1.020]	[−3.820, −2.560]	[−0.577, 0.736]	--	[−2.81, −2.18]	[−3.14, −2.62]
	
	**N_1_**	48	6	9	44	13	48	6	9	44	13
	
	**N_2_**	53	74	317	239	323	53	182	0	39	413

**Corrected (Effective) Sample Size**	**P-value**	2.31e-14	0.515	0.012	0.006	0.309	2.92e-05	0.897	--	5.65e-09	3.13e-10
	
	**T-statistic**	−15.2	1.07	−7.05	−3.36	1.19	−6.95	0.369	--	−8.92	−9.98
	
	**95% CI**	[−0.574, −0.438]	[−3.63, 3.94]	[−0.721, −0.221]	[−0.357, −0.075]	[−0.056, 0.131]	[−0.549, −0.285]	[−0.813, 0.906]	--	[−0.475, −0.294]	[−0.526, −0.341]
	
	**DF**	25.7	0.779	2.36	11.5	3.43	11.2	1.38	--	17	15.7
	
	**Hedges G**	−5.25	0.643	−2.46	−1.32	0.478	−3.25	0.222	--	−2.02	−2.05
	
	**Hedges G CI**	[−6.79, −3.71]	[−1.01, 2.30]	[−3.94, −0.987]	[−2.04, −0.606]	[−0.539, 1.49]	[−4.59, −1.90]	[−1.11, 1.56]	--	[−2.89, −1.15]	[−3.33, −0.760]
	
	**N_eff 1_**	15	2	2	10	4	8	2	2	7	3
	
	**N_eff 2_**	16	28	35	57	93	14	75	0	52	36

Two-sample two-tailed Welch’s t-tests (calculated using either the uncorrected sample size (top half of table) or the effective sample size (bottom half)) were conducted for the pre-DBS versus post-DBS periods using parameters of nonlinear autoregressive coefficient of determination ($R^{2}$). Abbreviations are the same as in Extended Data Table 1.

**Extended Data Table 4 |:** Daily sample entropy of 9-Hz VS power pre-DBS versus post-DBS in Cohort 1 patients

		Sample Entropy Left Hemisphere					Sample Entropy Right Hemisphere				
	
		B001	B002	B004	B005	B006	B001	B002	B004	B005	B006

**Uncorrected Sample Size**	**P-value**	4.10e-14	0.091	5.30e-5	1.43e-7	0.772	1.06e-19	0.799	--	3.57e-8	9.59e-11
	
	**T-statistic**	9.22	−1.98	6.48	5.98	0.296	12	0.265	--	6.15	13
	
	**95% CI**	[0.015, 0.023]	[−0.024, 0.002]	[0.010, 0.020]	[0.005, 0.010]	[−0.005, 0.007]	[0.018, 0.025]	[−0.010, 0.013]	--	[0.005, 0.010]	[0.014, 0.020]
	
	**DF**	78	6.59	10.7	58.8	13.5	81.4	6.75	--	74.2	18.5
	
	**Hedges G**	1.79	−0.825	1.53	0.993	0.092	2.33	0.089	--	0.908	2.06
	
	**Hedges G CI**	[1.31, 2.26]	[−1.69, 0.061]	[1.02, 2.03]	[0.644, 1.34]	[−0.517, 0.699]	[1.80, 2.85]	[−0.566, 0.742]	--	[0.604, 1.21]	[1.71, 2.41]
	
	**N_1_**	49	7	10	45	14	49	7	9	45	14
	
	**N_2_**	53	77	323	240	330	53	187	0	240	418

**Corrected (Effective) Sample Size**	**P-value**	1.55e-5	0.500	0.177	0.008	0.883	7.65e-7	0.901	--	0.008	2.19e-4
	
	**T-statistic**	5.29	−1.012	2.903	3.148	0.159	6.52	0.148	--	2.94	6.46
	
	**95% CI**	[0.012, 0.026]	[−0.157, 0.136]	[−0.030, 0.059]	[0.002, 0.012]	[−0.016, 0.018]	[0.015, 0.028]	[−0.059, 0.062]	--	[0.002, 0.013]	[0.011, 0.023]
	
	**DF**	26.1	0.970	1.20	13.3	3.11	25.2	1.33	--	21.6	7.79
	
	**Hedges G**	1.70	−0.97	1.26	1.03	0.119	2.18	0.080	--	0.839	1.61
	
	**Hedges G CI**	[0.864, 2.54]	[−2.66, 0.706]	[−0.191, 2.70]	[0.402, 1.65]	[−0.881, 1.12]	[1.20, 3.16]	[−1.30, 1.46]	--	[0.150, 1.53]	[0.559, 2.65]
	
	**N_eff 1_**	15	2	2	12	4	12	2	10	12	4
	
	**N_eff 2_**	18	16	65	96	133	17	58	0	41	48

Two-sample two-tailed Welch’s *t*-tests (calculated using either the uncorrected sample size (top half of table) or the effective sample size (bottom half)) were conducted for the pre-DBS versus post-DBS periods using parameters of sample entropy (per-day). Abbreviations are the same as in Extended Data Table 1.

**Extended Data Table 5 |:** Daily output measures of 9-Hz VS power pre-DBS versus post-DBS in Cohort 2 patients

			Uncorrected Sample Size								Corrected (Effective) Sample Size							
	
			P-value	T-statistic	95% CI	DF	Hedges G	Hedges G CI	N_1_	N_2_	P-value	T-statistic	95% CI	DF	Hedges G	Hedges G CI	N_eff 1_	N_eff 2_

**Left Hemisphere**	**Cosinor** $R^{2}$	**B007**	--	--	--	--	--	--	13	0	--	--	--	--	--	--	4	0
	
		**B008**	0.068	1.92	[−0.005, 0.126]	22.2	0.496	[−0.024, 1.01]	19	117	0.345	1.04	[−0.089, 0.210]	5.05	0.540	[−0.372, 1.45]	5	52
	
		**U001**	0.396	0.864	[−0.999, 2.43]	23.3	0.241	[−0.312, 0.789]	24	44	0.448	0.776	[−1.22, 2.66]	18.7	0.269	[−0.333, 0.870]	19	27

	**Linear AR** $R^{2}$	**B007**	--	--	--	--	--	--	13	0	--	--	--	--	--	--	4	0
	
		**B008**	0.452	−0.765	[−0.168, 0.077]	25.2	−0.182	[−0.650, 0.287]	19	117	0.771	−0.317	[−0.490, 0.398]	3.17	−0.173	[−1.41, 1.07]	3	20
	
		**U001**	0.012	−2.62	[−0.146, −0.019]	45.4	−0.661	[−1.17, −0.150]	24	44	0.157	−1.51	[−0.202, 0.037]	11.9	−0.654	[−1.59, 0.277]	7	17

	**Nonlinear AR** $R^{2}$	**B007**	--	--	--	--	--	--	13	0	--	--	--	--	--	--	4	0
	
		**B008**	0.853	−0.187	[−0.135, 0.113]	24.7	−0.045	[−0.518, 0.428]	19	117	0.943	−0.078	[−0.464, 0.441]	3.08	−0.043	[−1.27, 1.19]	3	21
	
		**U001**	0.606	−0.522	[−0.184, 0.109]	27.2	−0.142	[−0.676, 0.394]	24	44	0.714	−0.373	[−0.251, 0.177]	13.9	−0.156	[−0.896, 0.583]	12	20

	**Sample Entropy**	**B007**	--	--	--	--	--	--	14	0	--	--	--	--	--	--	4	0
	
		**B008**	0.630	0.487	[−0.004, 0.007]	25.6	0.117	[−0.355, 0.589]	20	118	0.828	0.228	[−0.013, 0.016]	5.39	0.115	[−0.956, 1.12]	4	20
	
		**U001**	0.167	1.40	[−0.001, 0.005]	67.0	0.320	[−0.134, 0.771]	25	44	0.496	0.699	[−0.004, 0.009]	15.0	0.283	[−0.801, 1.37]	6	11

**Right Hemisphere**	**Cosinor** $R^{2}$	**B007**	0.05	2.09	[−0.001, 0.239]	17.2	0.722	[0.008, 1.42]	13	29	0.419	0.983	[−0.348, 0.587]	2.26	0.780	[−0.828, 2.39]	3	9
	
		**B008**	0.126	1.60	[−0.025, 0.189]	19.3	0.472	[−0.125, 1.06]	19	95	0.390	0.928	[−0.136, 0.300]	5.82	0.661	[−0.235, 1.56]	6	30
	
		**U001**	0.451	−0.76	[−0.967, 0.437]	46.3	−0.161	[−0.575, 0.256]	24	119	0.492	−0.693	[−1.04, 0.508]	39.9	−0.134	[−0.756, 0.488]	15	37

	**Linear AR** $R^{2}$	**B007**	6.25e-8	−6.76	[−0.310, −0.167]	36.6	−1.82	[−2.49, −1.12]	13	29	0.025	−3.02	[−0.436, −0.042]	5.59	−1.50	[−3.29, 0.301]	4	6
	
		**B008**	0.605	0.523	[−0.102, 0.172]	25.1	0.132	[−0.362, 0.624]	19	95	0.775	0.297	[−0.243, 0.312]	7.03	0.132	[−0.768, 1.03]	6	31
	
		**U001**	0.127	−1.55	[−0.143, 0.018]	54.8	−0.378	[−0.86, 0.107]	24	119	0.454	−0.781	[−0.242 0.117]	9.17	−0.351	[−1.43, 0.730]	5	14

	**Nonlinear AR R^2^**	**B007**	5.22e-6	−5.30	[−0.271, −0.121]	38.1	−1.55	[−2.210, −0.873]	13	29	0.035	−2.67	[−0.386, −0.019]	6.34	−1.50	[−3.11, 0.099]	5	5
	
		**B008**	0.251	1.17	[−0.052, 0.192]	27.0	0.284	[−0.195, 0.761]	19	95	0.536	0.651	[−0.189, 0.332]	6.88	0.281	[−0.647, 1.21]	6	30
	
		**U001**	0.204	−1.29	[−0.144, 0.031]	55.9	−0.313	[−0.792, 0.168]	24	119	0.496	−0.702	[−0.231, 0.118]	12.6	−0.292	[−1.28, 0.696]	6	15

	**Sample Entropy**	**B007**	2.75e-8	7.03	[0.012, 0.022]	36.6	2.00	[1.30, 2.69]	14	34	0.014	3.26	[0.005, 0.030]	6.97	1.70	[−0.080, 3.48]	3	7
	
		**B008**	0.035	2.19	[0.000, 0.008]	35.4	0.477	[0.043, 0.908]	20	97	0.361	1.01	[−0.006, 0.015]	4.88	0.420	[−0.694, 1.53]	4	25
	
		**U001**	0.090	1.72	[−0.001, 0.007]	67.0	0.395	[−0.062, 0.849]	25	120	0.319	1.02	[−0.003, 0.010]	22.1	0.359	[−0.527, 1.24]	9	16

Two-sample two-tailed Welch’s *t*-tests (calculated using either the uncorrected sample size (left half of table) or the effective sample size (right half)) were conducted for the pre-DBS versus post-DBS periods using cosinor $R^{2}$, linear autoregressive $R^{2}$, nonlinear autoregressive $R^{2}$ and sample entropy (per-day) for the three Cohort 2 patients with both pre-DBS and post-DBS data. Patient B007 did not have post-DBS left hemisphere data due to an incompatible recording configuration. Patients B009, B010, U002 and U003 were not included in the table due to missing either pre-DBS or post-DBS data in both hemispheres. Abbreviations are the same as in Extended Data Table 1.

**Extended Data Table 6 |:** Comparison of symptom burdened versus unburdened states for all four metrics pooled across all patients

		Left Hemisphere				Right Hemisphere			
	
		Cosinor $R^{2}$	Linear AR $R^{2}$	Nonlinear AR $R^{2}$	Sample Entropy	Cosinor $R^{2}$	Linear AR $R^{2}$	Nonlinear AR $R^{2}$	Sample Entropy

**Uncorrected Sample Size**	**P-value**	0.008	9.68e-210	8.29e-195	1.60e-48	0.560	4.23e-34	3.20e-43	1.29e-18
	
	**T-statistic**	−2.67	−36.6	−34.9	15.2	0.583	−12.6	−14.5	9.05
	
	**95% CI**	[−0.125, −0.019]	[−0.383, 0.344]	[−0.389, 0.347]	[0.007, 0.009]	[−0.023, 0.043]	[−0.184, −0.134]	[−0.207, −0.158]	[0.006, 0.009]
	
	**DF**	798.2	1486.9	1472.2	1339.7	1102.0	1026.3	964.0	729.2
	
	**Hedges G**	−0.135	−1.88	−1.79	0.790	0.027	−0.701	−0.814	0.538
	
	**Hedges G CI**	[−0.235, −0.0358]	[−2.00, −1.76]	[−1.91, −1.67]	[0.684, 0.895]	[−0.064, 0.119]	[−0.812, −0.589]	[−0.929, −0.700]	[0.419, 0.656]
	
	**N_1_**	776	776	776	776	952	952	952	952
	
	**N_2_**	728	728	728	728	447	447	447	447

**Corrected (Effective) Sample Size**	**P-value**	0.078	3.25e-56	3.62e-50	4.44e-16	0.752	1.19e-9	4.03e-11	5.89e-6
	
	**T-statistic**	−1.77	−18.7	−17.5	8.48	0.317	−6.28	−6.94	4.64
	
	**95% CI**	[−0.153, 0.008]	[−0.402, −0.326]	[−0.409, −0.327]	[0.006, 0.010]	[−0.052, 0.071]	[−0.209, −0.109]	[−0.234, −0.131]	[0.004, 0.011]
	
	**DF**	354.9	389.9	365.8	408.8	343.7	293.4	228.7	225.7
	
	**Hedges G**	−0.125	−1.867	−1.757	0.794	0.023	−0.685	−0.798	0.551
	
	**Hedges G CI**	[−0.291, 0.041]	[−2.106, −1.628]	[−1.998, −1.516]	[0.605, 0.983]	[−0.205, 0.250]	[−0.915, −0.454]	[−1.04, −0.555]	[0.324, 0.777]
	
	**N_eff 1_**	342	213	224	244	288	204	210	203
	
	**N_eff 2_**	237	179	154	223	101	124	105	127

Two-sample two-tailed Welch’s *t*-tests (calculated using either the corrected sample size or the effective sample size) were conducted for the period in which patients had high symptom burden (that is, pre-DBS and persistent OCD symptoms post-DBS) versus the period with low symptom burden (that is, post-DBS clinical response) using cosinor $R^{2}$, linear autoregressive $R^{2}$, nonlinear autoregressive $R^{2}$ and sample entropy (per-day). Patient U002 was not included in the analysis due to incomplete data in the pre-DBS period. Abbreviations are the same as in Extended Data Table 1.

**Extended Data Table 7 |:** Leave-one-patient-out logistic regression classifier of all four metrics across all patients

			Left Hemisphere				Right Hemisphere			
	
			Cosinor	Linear AR	Nonlinear AR	Sample Entropy	Cosinor	Linear AR	Nonlinear AR	Sample Entropy

**AUROC**	**Delta**	**True Label**	0.665	0.851	0.894	0.776	0.121	0.562	0.600	0.245
	
		**Shuffled Label**	0.497	0.495	0.495	0.495	0.494	0.495	0.495	0.495
	
		**P-Val (random)**	0.0	0.0	0.0	0.0	1.0	7.0e-4	0.0	1.0
	
		**P-Val (circular)**	0.133	0.007	0.0	0.0	0.999	0.0	0.0	0.436

	**Daily**	**True Label**	0.712	0.877	0.870	0.671	0.268	0.588	0.594	0.477
	
		**Shuffled Label**	0.496	0.495	0.495	0.495	0.497	0.495	0.495	0.495
	
		**P-Val (random)**	0.0	0.0	0.0	0.0	1.0	0.0	0.0	0.749
	
		**P-Val (circular)**	5.0e-4	0.0	1.0e-4	0.0	0.920	0.0	0.0	0.0

**Balanced Accuracy**	**Delta**	**True Label**	0.688	0.821	0.835	0.692	0.265	0.613	0.656	0.365
	
		**Shuffled Label**	0.500	0.500	0.500	0.499	0.500	0.500	0.500	0.500
	
		**P-Val (random)**	0.0	0.0	0.0	0.0	1.0	0.0	0.0	1.0
	
		**P-Val (circular)**	0.008	0.0	0.0	0.100	0.814	0.005	2.0e-4	0.406

	**Daily**	**True Label**	0.689	0.819	0.805	0.647	0.321	0.606	0.594	0.581
	
		**Shuffled Label**	0.500	0.500	0.500	0.500	0.500	0.500	0.500	0.500
	
		**P-Val (random)**	0.0	0.0	0.0	0.0	1.0	0.0	0.0	0.0
	
		**P-Val (circular)**	0.070	0.0	0.0	0.0	0.840	1.0e-4	1.0e-4	0.0

Logistic regression models were trained to predict clinical status (symptom burdened versus unburdened state) based on either the daily VS measures (daily model) or the difference between that day’s measure and the average measure during the pre-DBS period (delta model). The daily model was trained on 11 patients, and the delta was trained on 10 patients. Class weights were adjusted to account for the class imbalances in the dataset. Leave-one-patient-out cross-validation was used to assess model performance using two performance metrics: AUROC and balanced accuracy. To determine significance, true test metrics were compared with chance distributions estimated through a permutation test (completed via random shuffling or circular shifting of labels). Significant *P* values indicate that the model performs better than chance. Patient U002 was not included in the analysis due to incmplete data in the pre-DBS period.

## Supplementary Material

video 1

video 2

suppl table 1

## Figures and Tables

**Table 1 |:** Patient demographics, clinical outcomes and duration of continuous neural recordings

Patient	Gender	Age at time of surgery (years)	Follow-up duration (months)	Responder status	Initial Y-BOCS II (Y-BOCS) score	Final Y-BOCS II (Y-BOCS) score	Percent Y-BOCS II reduction (Y-BOCS)	LFP recording duration days (L|R)
B001	Female	42	35	Yes	41 (40)	26 (24)	36.59% (40%)	150|150
B002	Female	31	13	No	44 (37)	42 (35)	4.55% (5.41%)	99|210
B004	Male	31	25	Yes	–(34)	21 (15)	–(55.88%)	411|11
B005	Male	20	20	Yes	42 (34)	14 (12)	66.67% (64.71%)	380|380
B006	Female	55	22	No	43 (38)	35 (31)	18.60% (18.42%)	344|432
B007	Male	23	19	Yes	35 (28)	9 (8)	74.29% (71.43%)	15|59
B008	Female	33	12	No^a^	36 (31)	26 (24)	27.78% (22.58%)	138|117
B009	Female	31	36	Yes	48 (38)	4 (4)	91.67% (89.47%)	120|120
B010	Female	21	1	No^a^	32 (30)	30 (26)	6.25% (13.33%)	24|24
U001	Female	33	4	No^a^	–(34)	–(31)	–(8.82%)	69|69
U002	Male	23	5	Yes^a^	–(30)	–(15)	–(50.00%)	16|30
U003	Female	24	1	No^a^	–(36)	–(34)	–(5.56%)	29|29

B001–B006 were included in Cohort 1 (*n* = 5). B007–B010 and U001–U003 were included in Cohort 2 (*n* = 7). Gender was determined based on self-report. Follow-up duration was calculated as the time between initial surgery and the most recent clinical visit. Responder status is defined as ≥35% reduction in the Y-BOCS. Patient B009 was implanted with the Percept device after undergoing a battery replacement, so the initial Y-BOCS-I and Y-BOCS-II scores are from the timepoint of the initial DBS surgery.

a Responder status is denoted by superscript letter if a patient had fewer than 6 months of follow-up.

## Data Availability

Data supporting the findings of this study are publicly available via the Data Archive for the BRAIN Initiative (DABI) registry (https://dabi.loni.usc.edu/dsi/B7KOT69PR2HP).
