# Author Correction: Disruption of neural periodicity predicts clinical response after deep brain stimulation for obsessive-compulsive disorder

**DOI:** 10.1038/s41591-025-03667-x

**Published:** 2025-03-26

**Authors:** Nicole R. Provenza, Sandesh Reddy, Anthony K. Allam, Sameer V. Rajesh, Nabeel Diab, Gabriel Reyes, Rose M. Caston, Kalman A. Katlowitz, Ajay D. Gandhi, Raphael A. Bechtold, Huy Q. Dang, Ricardo A. Najera, Nisha Giridharan, Katherine E. Kabotyanski, Faiza Momin, Mohammed Hasen, Garrett P. Banks, Brian J. Mickey, Brent M. Kious, Ben Shofty, Benjamin Y. Hayden, Jeffrey A. Herron, Eric A. Storch, Ankit B. Patel, Wayne K. Goodman, Sameer A. Sheth

**Affiliations:** 1 Department of Neurosurgery, Baylor College of Medicine, Houston, TX USA; 2 Department of Electrical & Computer Engineering, Rice University, Houston, TX USA; 3 Department of Neurosurgery, University of Utah, Salt Lake City, UT USA; 4 Department of Bioengineering, University of Washington, Seattle, WA USA; 5 Department of Psychiatry, University of Utah, Salt Lake City, UT USA; 6 Department of Biomedical Engineering, University of Utah, Salt Lake City, UT USA; 7 Department of Neuroscience, Baylor College of Medicine, Houston, TX USA; 8 Department of Neurological Surgery, University of Washington, Seattle, WA USA; 9 Menninger Department of Psychiatry and Behavioral Sciences, Baylor College of Medicine, Houston, TX USA

**Keywords:** Predictive markers, Anxiety, Cognitive control, Neural decoding

Correction to: *Nature Medicine* 10.1038/s41591-024-03125-0, published online 12 July 2024.

In the version of this article initially published, the statement of competing interests for Sameer A. Sheth did not mention consultancy for Abbott, as is now amended in the HTML and PDF versions of the article.

