# Supplementary material for: Disruption of neural periodicity predicts clinical response after deep brain stimulation for obsessive-compulsive disorder
Source: Nat Med. Author manuscript; Available in PMC 2024 Nov 4. (PMC11485242; doi:10.1038/s41591-024-03125-0)
Supplement: suppl table 1 [file NIHMS2019656-supplement-suppl_table_1.pdf]

	Left Hemisphere Pre-DBS Cosinor Fit						Right Hemisphere Pre-DBS Cosinor Fit					
	**R^2**	**P-value**	**F-statistic**	**DF**	**N**	**Comp-onents**	**R^2**	**P-value**	**F-statistic**	**DF**	**N**	**Comp-onents**
**B001**	0.376	0.0	691	6883	6890	3	0.074	1.08e-110	91.3	6883	6890	3
**B002**	0.290	1.06e-64	90.8	887	892	2	0.214	3.07e-45	60.5	887	892	2
**B004**	0.361	6.45e-120	346	1227	1230	1	0.165	6.81e-49	122	1227	1230	1
**B005**	0.110	1.21e-157	383	6226	6229	1	0.143	4.18e-209	519	6226	6229	1
**B006**	0.322	2.31e-153	429	1811	1814	1	0.415	8.94e-212	643	1811	1814	1

Supplemental Table 1: Strength of neural circadian rhythms in the severe OCD symptom state before DBS in Cohort 1. A single cosinor (regression model for sum of cosines) was fitted to the 9 Hz power data in VS over the entire pre-DBS period. We computed the coefficient of determination (R^2) and compared the fit to a null model via a two-sided general linear F-test to determine significance. The sample size (N) denoted here represents the number of power values (9 Hz frequency band) collected at 10-minute intervals from the DBS device. The number of cosinor components reflects the maximum number of daily peaks in 9 Hz power (see Methods). Abbreviations: R^2, coefficient of determination; VS, ventral striatum; N, sample size; DF, degrees of freedom

<table>
<tr><th colspan="2" rowspan="2"></th><th colspan="4">Left Hemisphere</th><th colspan="4">Right Hemisphere</th></tr>
<tr><th>Cosinor</th><th>Linear AR</th><th>Nonlinear AR</th><th>Sample Entropy</th><th>Cosinor</th><th>Linear AR</th><th>Nonlinear AR</th><th>Sample Entropy</th></tr>
<tr><th rowspan="4">Daily Model P-value (Z statistic)</th><th>Cosinor</th><td>--</td><td>1.18e-40 (-13.3)</td><td>6.81e-38 (-12.9)</td><td>0.060 (1.88)</td><td>--</td><td>3.37e-50 (-14.9)</td><td>6.54e-49 (-14.7)</td><td>2.95e-19 (-8.97)</td></tr>
<tr><th>Linear AR</th><td>--</td><td>--</td><td>0.003 (3.03)</td><td>2.95e-52 (15.2)</td><td>--</td><td>--</td><td>0.251 (-1.15)</td><td>4.08e-11 (6.60)</td></tr>
<tr><th>Nonlinear AR</th><td>--</td><td>--</td><td>--</td><td>7.61e-48 (14.5)</td><td>--</td><td>--</td><td>--</td><td>2.071827 30919783 e-13 (7.34)</td></tr>
<tr><th>Sample Entropy</th><td>--</td><td>--</td><td>--</td><td>--</td><td>--</td><td>--</td><td>--</td><td>--</td></tr>
<tr><th rowspan="4">Delta Model P-value (Z statistic)</th><th>Cosinor</th><td>--</td><td>1.94e-38 (-13.0)</td><td>5.51e-54 (-15.5)</td><td>4.58e-12 (-6.92)</td><td>--</td><td>2.42e-166 (-27.5)</td><td>3.10e-200 (-30.2)</td><td>5.83e-13 (-7.20)</td></tr>
<tr><th>Linear AR</th><td>--</td><td>--</td><td>7.04e-18 (-8.61)</td><td>1.02e-09 (6.11)</td><td>--</td><td>--</td><td>4.56e-19 (-8.92)</td><td>6.98e-57 (15.9)</td></tr>
<tr><th>Nonlinear AR</th><td>--</td><td>--</td><td>--</td><td>2.97e-23 (9.93)</td><td>--</td><td>--</td><td>--</td><td>1.85e-76 (18.5)</td></tr>
<tr><th>Sample Entropy</th><td>--</td><td>--</td><td>--</td><td>--</td><td>--</td><td>--</td><td>--</td><td>--</td></tr>
</table>

Supplemental Table 2: DeLong test statistics for pairwise comparisons of leave-one-patient-out classifier performance. The DeLong test statistic was calculated between pairwise comparisons of the ROC curve of the four logistic regression models trained to predict clinical status (i.e., daily or delta). A positive z-statistic indicates that the model denoted by the row has a greater classification performance compared to the model denoted by the column. P-values below 0.05 denote statistically significant differences between ROC curves and are highlighted in light green.
