# Supplementary material for: Disruption of neural periodicity predicts clinical response after deep brain stimulation for obsessive-compulsive disorder
Source: Nat Med. 2024 Jul 12;30(10):3004–14. doi: 10.1038/s41591-024-03125-0 (PMC11485242; doi:10.1038/s41591-024-03125-0)
Supplement: Supplementary file 2 [file 41591_2024_3125_MOESM2_ESM.pdf]

Corresponding author(s): Sameer A. Sheth, MD, PhD

Last updated by author(s): 6/1/2024

Reporting Summary

Nature Portfolio wishes to improve the reproducibility of the work that we publish. This form provides structure for consistency and transparency in reporting. For further information on Nature Portfolio policies, see our Editorial Policies and the Editorial Policy Checklist.

Statistics

For all statistical analyses, confirm that the following items are present in the figure legend, table legend, main text, or Methods section.

n/a	Confirmed	
☐	☒	The exact sample size (*n*) for each experimental group/condition, given as a discrete number and unit of measurement
☐	☒	A statement on whether measurements were taken from distinct samples or whether the same sample was measured repeatedly
☐	☒	The statistical test(s) used AND whether they are one- or two-sided<br>*Only common tests should be described solely by name; describe more complex techniques in the Methods section.*
☐	☒	A description of all covariates tested
☐	☒	A description of any assumptions or corrections, such as tests of normality and adjustment for multiple comparisons
☐	☒	A full description of the statistical parameters including central tendency (e.g. means) or other basic estimates (e.g. regression coefficient) AND variation (e.g. standard deviation) or associated estimates of uncertainty (e.g. confidence intervals)
☐	☒	For null hypothesis testing, the test statistic (e.g. F, t, r) with confidence intervals, effect sizes, degrees of freedom and P value noted<br>*Give P values as exact values whenever suitable.*
☒	☐	For Bayesian analysis, information on the choice of priors and Markov chain Monte Carlo settings
☒	☐	For hierarchical and complex designs, identification of the appropriate level for tests and full reporting of outcomes
☒	☐	Estimates of effect sizes (e.g. Cohen's *d*, Pearson's *r*), indicating how they were calculated

Our web collection on statistics for biologists contains articles on many of the points above.

Software and code

Policy information about availability of computer code

Data collection	Data were collected through the Medtronic DBS Clinician Programmer Application. The clinic visit information was exported from the tablet as a de-identified JSON and PDF report.
Data analysis	Data analysis was conducted on MATLAB 2022b and Python 3.10.11. MATLAB analysis included the Signal Processing and EntropyHub (v0.2) toolboxes. Python analysis included the statsmodel 0.14.0 and scikit-learn 1.2.2 packages. The custom algorithms and code for all the data analyses are available on the GitHub repository [https://github.com/shethlab/PerceptDataAnalysis].

For manuscripts utilizing custom algorithms or software that are central to the research but not yet described in published literature, software must be made available to editors and reviewers. We strongly encourage code deposition in a community repository (e.g. GitHub). See the Nature Portfolio guidelines for submitting code & software for further information.

Data

Policy information about availability of data

All manuscripts must include a data availability statement. This statement should provide the following information, where applicable:

- Accession codes, unique identifiers, or web links for publicly available datasets
- A description of any restrictions on data availability
- For clinical datasets or third party data, please ensure that the statement adheres to our policy

Raw data were generated at Baylor College of Medicine and University of Utah during psychiatric clinic visits. Data supporting the findings of this study are publicly available via the DABI registry (linked here: https://dabi.loni.usc.edu/dsi/B7KOT69PR2HP).

Human research participants

Policy information about studies involving human research participants and Sex and Gender in Research.

Reporting on sex and gender	Consent was obtained to report gender for each study participant. Gender was determined based on self-report. Neither sex nor gender was considered in the study design, and sex/gender analyses was not carried out due to the small sample size and lack of pre-existing hypotheses on sex/gender differences.
Population characteristics	All patients had a primary diagnosis of treatment-resistant OCD and were implanted in the VCVS. Four of these patients had a co-primary diagnosis of Tourette's syndrome and were dual-implanted in the GPi. Four are male, eight are female. Ages at surgery range from 20 to 55.
Recruitment	Study participation was offered to every patient undergoing DBS for OCD with a Percept IPG. 12 out of 12 subjects undergoing this procedure during the study period enrolled. Recruitment was facilitated by large OCD management programs in the Texas Medical Center and utilized careful monitoring of referral streams, advertising in key clinics and hospitals, and partnering with the International OCD Foundation and other patient resources. Since participation in the research was offered to every patient and all patients agreed, there is no bias introduced by the research. However, we cannot rule out any bias in the clinical population that is getting these procedures.
Ethics oversight	Each participant gave fully informed consent. The protocol was approved by the local institutional review boards at Baylor College of Medicine (Protocol number 48392) and University of Utah (Protocol number 00169174).

Note that full information on the approval of the study protocol must also be provided in the manuscript.

Field-specific reporting

Please select the one below that is the best fit for your research. If you are not sure, read the appropriate sections before making your selection.

☒ Life sciences ☐ Behavioural & social sciences ☐ Ecological, evolutionary & environmental sciences

For a reference copy of the document with all sections, see nature.com/documents/nr-reporting-summary-flat.pdf

Life sciences study design

All studies must disclose on these points even when the disclosure is negative.

Sample size	The sample sized was determined based on the number of patients implanted at Baylor College of Medicine and University of Utah. No sample-size calculation was performed. Analyses were performed both within and across subjects with leave-one-patient out analysis. The cross-validated results were strongly significant. The sample size of 12 is greater than most other intracranial studies in psychiatric patients.
Data exclusions	None of the data were excluded.
Replication	Our full sample consisted of two patient cohorts. Cohort 1 consisted of the first 5 individuals (B001, B003, B004, B005, and B006), from whom we have the longest recordings, starting before DBS initiation and spanning months afterwards until they achieved stable chronic clinical status. Data from these individuals were used to construct the neurobehavioral models. Cohort 2 consisted of the next 7 participants (B007-U003), whose data were used to replicate/validate the findings. The attempt to replicate the findings in Cohort 2 was successful.
Randomization	Randomization is not relevant to our study design as clinical outcomes of the intervention was not the primary focus, but rather the neurobehavioral relationship. Participants were not allocated into experimental groups. Covariates such as gender and age would not be expected to affect the neurobehavioral relationships resulting from this study.
Blinding	Blinding is not relevant to our study design because we are not testing the effect of the intervention on clinical outcomes.

Reporting for specific materials, systems and methods

We require information from authors about some types of materials, experimental systems and methods used in many studies. Here, indicate whether each material, system or method listed is relevant to your study. If you are not sure if a list item applies to your research, read the appropriate section before selecting a response.

Materials & experimental systems

n/a	Involved in the study
☒	☐ Antibodies
☒	☐ Eukaryotic cell lines
☒	☐ Palaeontology and archaeology
☒	☐ Animals and other organisms
☐	☒ Clinical data
☒	☐ Dual use research of concern

Methods

n/a	Involved in the study
☒	☐ ChIP-seq
☒	☐ Flow cytometry
☒	☐ MRI-based neuroimaging

Clinical data

Policy information about clinical studies

All manuscripts should comply with the ICMJE guidelines for publication of clinical research and a completed CONSORT checklist must be included with all submissions.

Clinical trial registration	NCT05915741
Study protocol	The full clinical trial protocol can be accessed online at clinicaltrials.gov
Data collection	Data collection was initiated at Baylor College of Medicine and University of Utah in the clinic and continued in the natural environments of subjects from November 2020 to January 2024.
Outcomes	0: <25% reduction in Y-BOCS scores (non-response), 1: 25-35% Y-BOCS score reduction (partial response), 2: ≥35% Y-BOCS score reduction (response), 3: ≥35% Y-BOCS score reduction and final Y-BOCS ≤ 12 (remitter). These measures were predefined based on the standard clinical measures for OCD, and assessed periodically (approximately monthly) throughout treatment.
